# Quantification of N-terminal amyloid-β isoforms reveals isomers are the most abundant form of the amyloid-β peptide in sporadic Alzheimer’s disease

**DOI:** 10.1093/braincomms/fcab028

**Published:** 2021-03-09

**Authors:** Soumya Mukherjee, Keyla A Perez, Larissa C Lago, Stephan Klatt, Catriona A McLean, Ian E Birchall, Kevin J Barnham, Colin L Masters, Blaine R Roberts

**Affiliations:** 1 Florey Institute of Neuroscience and Mental Health, University of Melbourne, Melbourne, VIC 3010, Australia; 2 Department of Anatomical Pathology, Alfred Hospital, Prahran, VIC 3004, Australia; 3 Department of Biochemistry, Emory University School of Medicine, Atlanta, GA 30322, USA; 4 Department of Neurology, Emory University School of Medicine, Atlanta, GA 30322, USA

**Keywords:** Alzheimer’s disease, amyloid-β, peptide, long-lived peptide, isomerization, mass spectrometry

## Abstract

Plaques that characterize Alzheimer’s disease accumulate over 20 years as a result of decreased clearance of amyloid-β peptides. Such long-lived peptides are subjected to multiple post-translational modifications, in particular isomerization. Using liquid chromatography ion mobility separations mass spectrometry, we characterized the most common isomerized amyloid-β peptides present in the temporal cortex of sporadic Alzheimer’s disease brains. Quantitative assessment of amyloid-β N-terminus revealed that > 80% of aspartates (Asp-1 and Asp-7) in the N-terminus was isomerized, making isomerization the most dominant post-translational modification of amyloid-β in Alzheimer’s disease brain. Total amyloid-β_1–15_ was ∼85% isomerized at Asp-1 and/or Asp-7 residues, with only 15% unmodified amyloid-β_1–15_ left in Alzheimer’s disease. While amyloid-β_4–15_ the next most abundant N-terminus found in Alzheimer’s disease brain, was only ∼50% isomerized at Asp-7 in Alzheimer’s disease. Further investigations into different biochemically defined amyloid-β-pools indicated a distinct pattern of accumulation of extensively isomerized amyloid-β in the insoluble fibrillar plaque and membrane-associated pools, while the extent of isomerization was lower in peripheral membrane/vesicular and soluble pools. This pattern correlated with the accumulation of aggregation-prone amyloid-β_42_ in Alzheimer’s disease brains. Isomerization significantly alters the structure of the amyloid-β peptide, which not only has implications for its degradation, but also for oligomer assembly, and the binding of therapeutic antibodies that directly target the N-terminus, where these modifications are located.

## Teaser

Mukherjee *et al.* report more than 80% of amyloid β N-terminus accumulating in sporadic Alzheimer’s disease is isomerized at Asp-1 and Asp-7 using ion mobility mass spectrometry. The discovery has implications for drug discovery, particularly immunotherapeutic strategies that often target the N-terminus of the peptide.

## Graphical Abstract

**Graphical Abstract:**
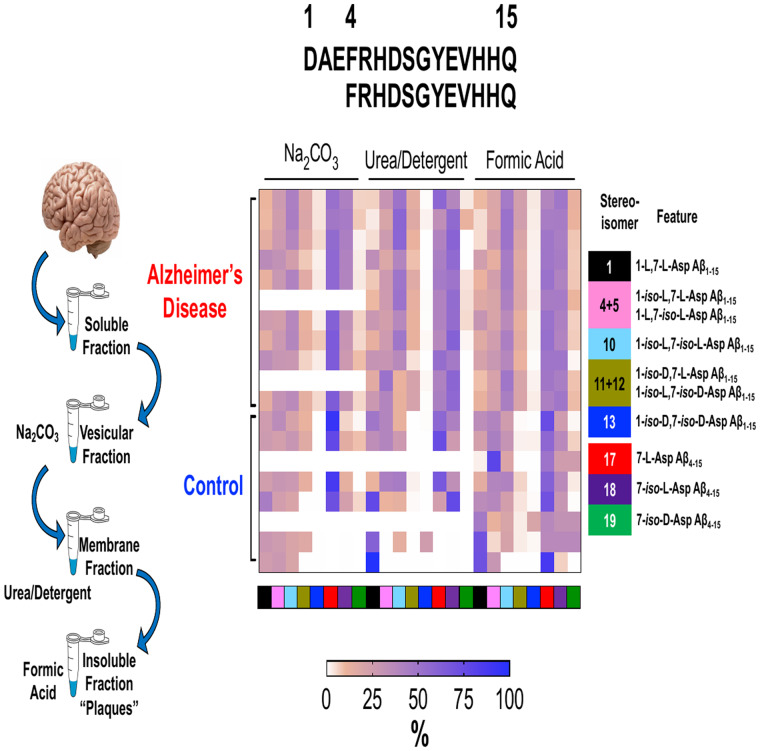

## Introduction

Neuropathology and amyloid-β (Aβ) positron emission tomography (PET) studies indicate that the accumulation of Aβ in sporadic Alzheimer’s disease brain begins more than 20 years before the onset of clinical symptoms.^1^^,^^2^ Evidence supports that this accumulation is a result of decreased clearance and not a change in the production of Aβ in sporadic Alzheimer’s disease.^3^^,^^4^ The subtle 2–5% decrease in its clearance results in total accumulation of ∼6.5 mg Aβ in the brain over the 20 year time span^2^^,^^5^ compared to 1.7 mg in age-matched control tissue. However, several questions regarding the Aβ-amyloid hypothesis^6^ remain unanswered, including what leads to the decrease in clearance and what triggers the aggregation of Aβ into extracellular plaques^7^ along with intracellular tau-reactive neurofibrillary tangles.^8^ The impairment in the clearance increases the half-life of the Aβ polypeptide and the process of amyloidosis in Alzheimer’s disease entombs the peptide for decades, making it a long-lived peptide. The prolonged time frame of amyloidosis is a common feature across multiple neurodegenerative diseases,^9^^,^^10^ predisposing the polypeptide chains to undergo multiple spontaneous non-enzymatic post-translational modifications (PTMs), which can render them resistant to normal cellular proteolysis mechanisms.^11^^,^^12^

**Figure 1:**
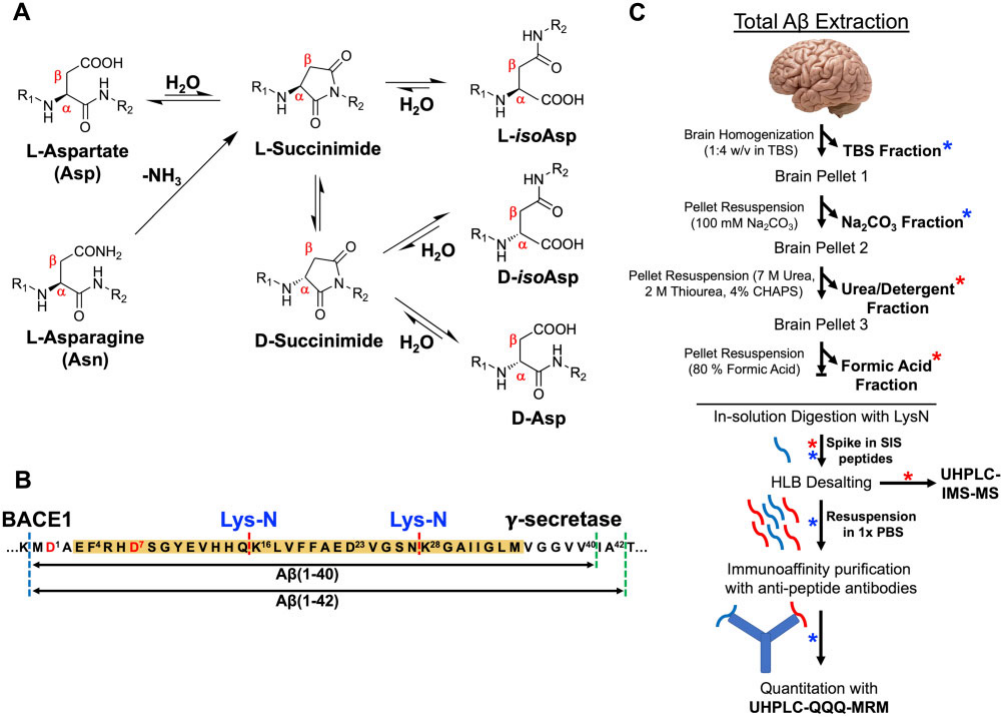
**Total amyloid-β extraction from human brain.** (**A**) Schematic representation of the spontaneous mechanism of dehydration of l-Asp as well as deamidation of l-Asn forming a succinimide intermediate that subsequently leads to the isomerization/racemization after ring opening to d/l-iso-Asp and d-Asp. (**B**) Amyloidogenic processing of amyloid precursor protein (APP) by β-secretase (dashed blue line) and γ-secretase (dashed green lines) leads to generation of canonical Aβ_1–40_ and Aβ_1–42_ peptides. Proteolytic digestion using LysN enzyme (dashed red line) of the Aβ peptide generates N-terminal, mid-domain and C-terminal fragments that were probed for quantitative evaluation in this study. (**C**) Quantitative proteomics workflow for the estimation of total Aβ in the amyloid rich biochemical pools of the brain after digestion with LysN enzyme and spiking of respective stable isotope standard (SIS) peptides without any enrichment strategy (red asterisk). SISCAPA strategy was used for the sparsely enriched peripheral/vascular (Na_2_CO_3_) pool and soluble pool (TBS) of Aβ after enzymatic digestion with LysN and spiking with SIS peptides (indicated by blue asterisk).

The earliest Edman sequencing and more recent mass spectrometry-based analyses have shown that there is a diverse population of N-terminally truncated species of Aβ_42_ (e.g. Aβ_1__–__42_, Aβ_2__–__42_, Aβ_4__–__42_).^7^^,^^13^ Moreover, multiple PTMs of Aβ have been described and include nitration,^14^ pyroglutamate formation,^15^^,^^16^ phosphorylation,^17^ methionine oxidation,^18^ dityrosine cross-linking^19^ and structural changes of the polypeptide backbone. Structural changes, in particular, occur on the amino acid level via non-enzymatic, spontaneous processes and facilitated by the amino acids with asymmetric central carbon atom. The most common structural protein modification associated with aging is stereoisomerization of Asp/Asn (aspartate/asparagine) residues and have been particularly useful for protein dating.^20^^,^^21^ Deamidation of l-Asn residue to l-Asp as well as racemization/isomerization to d-Asp and d/l-*iso*-Asp via succinimide intermediate^22^ (Fig. 1A) potentially should provide the information of the age of the Aβ plaques.^23^ In Alzheimer’s disease brain, the striking feature of the fibrillar Aβ is its sequential N-terminal truncation along with Asp and Ser (serine) epimerization.^13^^,^^15^^,^^24^^,^^25^ Qualitative estimates from the plaque-derived Aβ indicate almost 25% of Asp-1 and 75% Asp-7 are isomerized in Alzheimer’s disease brains.^9^^,^^26–29^

The antibodies currently in clinical trials target multiple forms (soluble oligomeric and insoluble fibrils) of Aβ due to their potential roles in the pathogenesis and disease progression.^30^^,^^31^ Other than the mid-domain and C-terminus Aβ, the other most common target epitope of these antibodies is the PTM-prone N-terminus of Aβ.^32–34^ In-depth understanding of the target engagement warrants detailed analysis of the PTMs (especially isomerization) associated with these epitopes. However, comprehensive characterization of these isomers/epimers along with their quantitative estimation is yet to be done in Alzheimer’s disease brain compared to age-matched control brains.^29^ Identification and quantification of the most relevant stereoisomers/structural isomers of Aβ is challenging. These isomers are structurally similar, which increases the difficulty of chromatographic separation and are indistinguishable to single-stage mass spectrometers (MS) due to their identical mass-to-charge (*m/z*) ratios. Analytical chromatographic separation of N-terminal isomers and epimers of Aβ and their simultaneous characterization using MS/MS fragmentation techniques have been investigated.^35–39^ Chiral chromatography was shown to separate synthetic Aβ epimers and isomers containing Asp and Ser residues.^40^ Ion mobility separation-mass spectrometry (IMS-MS) is a powerful tool for the analysis and characterization of isomerized and epimerized peptides in the gas phase.^41–45^ Recently, synthetic tryptic Aβ peptide isomers have been shown to resolve in IMS-MS using structures for lossless ion manipulations (SLIM).^46^^,^^47^ Coupling of online LC to SLIM-IMS demonstrated the potential of LC-IMS-MS in resolving challenging peptide isomers.^47^

In this article, we have identified, characterized and quantified the most common isomers of Aβ isoforms extracted from the temporal cortex of Alzheimer’s disease brains by using liquid chromatography (LC) coupled to drift tube IMS-QTOF MS. In particular, we determined Alzheimer’s disease-specific changes of the Aβ N-terminal pool in comparison to age-matched control brains; we report the total levels of Aβ_1__–__15_ and its associated modified isomers. We also quantified the total levels of the most abundant isomers of Aβ_4__–__15_. As an extension of the previously reported biochemical method,^5^ we have further quantified the amount of the two classical C-terminal isoforms of Aβ, i.e. Aβ_42_ and Aβ_40_^13^ in the most Aβ-enriched brain compartments. More than 92% of Aβ in *post-mortem* brains is partitioned in the insoluble/fibrillar and detergent soluble phase, while only <7% is extractable as vesicular and soluble.^5^ For quantitative estimation of Aβ peptides in these moderately/sparsely enriched pools, we developed stable isotope standards and capture by anti-peptide antibodies (SISCAPA)^48^^,^^49^ of Aβ with polyclonal antibodies. The distribution of the isomer ratios indicated a pattern of compartmentalization of highly isomerized Aβ_1__–__15_ and Aβ_4__–__15_ in the insoluble/fibrillar and membrane pool, with a comparatively lower extent of isomerization in the vesicular and soluble pools. This data allowed us to estimate the accurate biochemical identity and distribution of the spontaneously isomerized Aβ in post-mortem brain. This PTM is intricately associated with slow turnover rates and degradation of Aβ which accumulates over decades in sporadic Alzheimer’s disease.

## Materials and methods

All LC-MS grade solvents, acetonitrile (ACN), formic acid (FA), trifluoroacetic acid (TFA), acetic acid, isopropanol and urea, thiourea, N, N-Bis(2-hydroxyethyl)glycine (Bicine), 3-[(3-Cholamidopropyl)dimethylammonio]-1-propanesulfonate hydrate (CHAPS), iodoacetamide (IAA), tri-ethyl ammonium bicarbonate buffer, NaCl, Na_2_CO_3_, Tris buffers were purchased from Merck-Sigma or ThermoFischer Scientific. EDTA-free protease inhibitors from Roche. Bond-Breaker_**™**_ tris(2-carboxyethyl)phosphine (TCEP) Solution, neutral pH was from ThermoFischer Scientific. MS-grade metalloprotease LysN from *Grifola frondosa*, and dithiothreitol (DTT) were purchased from ThermoFischer Scientific. Biomasher were purchased from Omni International. The MS vials, Advanced Bio Peptide Mapping C_18_ Column (2.1 × 150 mm, 2.7 µm) and ESI low concentration tune mix used for instrument calibration were obtained from Agilent Technologies (Santa Clara, USA). Oasis HLB µElution 96 well-plates were purchased from Waters. Affinity purification was performed using PureProteome NHS FlexiBind Magnetic Beads from Millipore. Stable isotope standards (SIS) of Aβ peptides DAEF(R + 10)HDSGYEVHHQ, F(R + 10)HDSGYEVHHQ, (K + 8)GAIIGLMVGGVV, (K + 8)GAIIGLMVGGVVIA and K(+8)LVFFAEDVGSN were purchased from New England Peptides (MA, USA) and their concentration determined by amino acid analysis. Stock solutions of SIS Aβ peptides were prepared in 2% ACN, 0.05% TFA to a final concentration of 200 fmol/µL and stored at −20˚C. All the isomerized Aβ peptide standards were commercially synthesized and purchased from JPT Peptide Technologies (Germany). All the SIS isomeric Aβ peptides were resuspended in 30% ACN, 0.1% FA at 0.2 nmol/µL which were subsequently diluted to ∼2 pmol/µL in 15% ACN, 0.1% FA and stored at −20˚C.

### Brain tissue

Twenty post-mortem temporal cortex tissue samples were obtained from the Victorian Brain Bank (Australia). In detail, the cohort consisted of age-matched healthy control brains (*n* = 9), where the number of plaques and tangles were histopathologically analyzed and well below the cut-off values for Alzheimer’s disease. No other major neuropathological disease was present. Alzheimer’s disease brains (*n* = 11) met the standard criteria for Alzheimer’s disease neuropathological diagnosis (Demographic summary Supplementary Table 1). The study followed the ethics committees of the University of Melbourne (Ethics 1750801.3).

### Immunohistochemistry

Segments of frontal cortex from the same cases were fixed in 10% neutral buffered formalin and processed by standard histological methods for paraffin embedding and sectioning (8 µm). Sections were deparaffinised, endogenous peroxidase blocked with 5% aqueous hydrogen peroxide (5 min), treated (5 min) with 98–100% FA (Scharlau AC10852500), rinsed and immersed in Tris buffer (0.5 M pH 7.6). Sections were incubated in a 1/100 dilution of Dako anti-amyloid antibody (MO872—clone 6 F/3D) for 60 min at room temperature. Positively labelled Aβ was detected with a peroxidase labelled streptavidin/biotin system (Dako K0675) with a diaminobenzidine chromogen. Sections were counterstained with Harris’s haematoxylin, dehydrated and cover-slipped for imaging. Low and high magnification images were obtained with a Leica ICC50 HD camera on a Leica DM 750 binocular microscope.

### Tissue homogenization protocol and fractionation

Hemisected freshly frozen post_**-**_mortem brain tissue was processed as previously described with some modifications.^5^ Briefly, the frozen brains at −80°C were warmed to −20°C on ice and the leptomeningeal vessels were removed. The grey matter was dissected into ∼0.25 g aliquots from temporal cortex (Brodmann’s area 21). During dissection process, care was taken to keep the tissues frozen. The tissue was weighed out and was first bio-mashed through the Biomasher (Omni International) by centrifugation at 14 000 *g* for 1 min at room temperature. To the bio_**-**_mashed tissue, Tris-buffered saline (TBS, 50 mM Tris–HCl, 150 mM NaCl, pH 8.5) containing EDTA-free protease inhibitors (Roche) was added at a ratio of 1:4 (tissue: buffer, w/v). This solution was transferred to ultracentrifuge tubes (Beckman Coulter) and centrifuged (Optima MAX-XP from Beckman Coulter) at 100 000 *g* for 30 min at 4°C. The supernatant was collected, referred to as TBS fraction henceforth, and stored on ice until freezing.

The resulting pellet was then resuspended in 100 mM Na_2_CO_3_ pH 11 (1:4, tissue: buffer) and incubated for 20 min on ice before another ultracentrifugation step at 100 000 *g* was carried out for 30 min at 4°C. The supernatant containing peripheral membrane and vesicular material was recovered into an Eppendorf tube, referred to as Na_2_CO_3_ fraction.

The pellet resulting from Na_2_CO_3_ fractionation was resuspended with urea_**−**_detergent buffer (7 M urea, 2 M thiourea, 4% CHAPS, 30 mM bicine, pH 8.5) and spun at 100 000 *g* for 30 min at 4°C. The supernatant was aspirated out, referred to as urea_**−**_detergent fraction. These three biochemical fractions were then snap frozen in liq. N_2_ and stored at −80°C until further processing.

The residual pellet was finally incubated in 200 µL 70% glass-distilled FA (GDFA) for 2 h at room temperature in a fume hood. The FA fractions were spun at 13 200 *g* for 15 min at 4°C and supernatant was collected. The FA fractions (fourth biochemical fraction) were aliquoted into 10 µL portions and snap frozen in liquid N_2_, freeze dried in a lyophilizer and stored at −80°C. A summary of the biochemical fractionation procedure can be found in Fig. 1C.

### In-solution LysN digestion of formic acid, urea–detergent, Na_2_CO_3_ and TBS fractions

A total of 10 µL of both lyophilized FA and urea_**−**_detergent fractions were re_**−**_suspended/diluted to 100 µL in 100 mM tri-ethyl ammonium bicarbonate buffer (TEAB), pH 8.5. Next, the samples were reduced by incubating with dithiothreitol (DTT) to a final concentration of 20 mM at 37°C for 30 min, followed by alkylation using 25 mM iodoacetamide (IAA) in the dark for another 30 min. The samples were then diluted to 200 µL with 100 mM TEAB buffer, pH 8.5 and digested overnight by incubation at 37°C after adding LysN metalloprotease at enzyme: protein ratio of 1:100. The same in-solution digestion process was performed with 50 µL for the Na_2_CO_3_ and 100 µL for the TBS fractions. The Na_2_CO_3_ fraction was diluted to 100 µL and the TBS fraction to 170 µL with 8 M urea, 100 mM TEAB buffer (pH 8.5), respectively. Sample reduction and alkylation were carried out as described above. Finally, the two fractions were diluted to 250 µL for proteolytic digestion with LysN. All the proteomic sample processing was performed at pH 8.5. The digestion reaction was quenched by adding 10% FA to a final concentration of 0.1%. The FA and urea_**−**_detergent samples were then spiked with 10 µL of SIS Aβ peptides mixture (200 fmol/µL of Aβ NEP peptides), while only 5 µL was spiked into the Na_2_CO_3_ and TBS samples. The acidified samples were finally loaded onto an Oasis HLB µElution 96 well-plate (Waters). The wells were washed with 250 µL of 0.1% FA, followed by 250 µL of 5% methanol, 0.1% FA. The peptides were finally eluted with two sequential washes of 25 µL of 75% ACN, 0.1% FA. The eluent was lyophilized and stored at −20°C until further processing. The FA and urea_**−**_detergent samples were re-constituted in 25 µL of 2% ACN, 0.05% TFA, vortexed for 30 min on ice and sonicated for 2 min. The re-constituted samples were centrifuged at 10 000 *g* for 5 min and the supernatant was aliquoted in MS vials (Agilent Technologies) for analysis.

### Generation of anti-peptide antibodies

An integrated commercial procedure (New England Peptides, MA, USA) was used to generate affinity purified rabbit polyclonal antibodies against Lys-N cleaved Aβ_1__–__40_ and Aβ_1__–__42_ peptide sequences (Fig. 1B), i.e. Aβ_1__–__15_, Aβ_4__–__15_, Aβ_16__–__27_, Aβ_28__–__40_ and Aβ_28__–__42_. The lyophilized antibodies were re-constituted in 0.05% azide solution at ∼1 mg/mL with shaking for 1 h on ice and aliquoted into vials to avoid repeated freeze-thaw cycles and stored at −80°C.

### Aβ SISCAPA (stable isotope standards and capture by anti-peptide antibodies)

Enrichment experiments were performed in a round-bottom 96-well polypropylene plates using the magnetic bead protocol. The NEP Aβ anti-peptide antibodies were coupled to PureProteome NHS FlexiBind Magnetic Beads (Millipore) according to the manufacture’s protocol. At first, the capture efficiencies of the anti-peptide antibodies were determined in a complex background. Lyophilized LysN-digested pooled brain homogenate (10 µg total digested protein) was resuspended in 200 µL with PBS, 0.03% CHAPS pH 7.5 buffer along with the 500 fmol of respective SIS Aβ peptides and 1 µg of specific antibody (Supplementary Fig. 1). These antibodies specifically captured LysN-cleaved versions of Aβ peptides with no cross-reactivity for tryptic-cleaved versions.

For the multiplexed experiment, 1 µg of each antibody was added to the sample mixture and 1 M Tris–HCl pH 7.5 to a final concentration of 0.2 mM. To this mixture, 500 fmol SIS Aβ peptides were added. The mixture was incubated overnight at 4°C with shaking at 800 rpm. After overnight incubation the magnetic beads were magnetized, and the supernatant was discarded. Next, the magnetic beads were manually washed three times with 0.1 M ammonium acetate, 0.5 M NaCl, 0.03% CHAPS (pH 7.5) followed by another three washes with 0.1 M ammonium acetate, 15% ACN, pH 7.5. Finally, the captured peptides were eluted from the magnetic beads with 25 µL of 5% acetic acid, 15% ACN with shaking at 600 rpm and 2 min incubation.

This SISCAPA process was used only on the LysN-digested lyophilized Na_2_CO_3_/TBS brain fractions for Aβ enrichment.

### LC-drift tube ion mobility mass spectrometry

An Agilent 1290 Infinity series UHPLC system coupled to Agilent 6560 Drift Tube Ion Mobility QToF high-resolution MS (Agilent Technologies, Santa Clara, USA) was used for UHPLC-ESI-IM-MS separations. 0.1% FA in water (mobile phase A) and 0.1% FA in 100% ACN (mobile phase B) were used as a solvent system. Samples were loaded onto an Agilent Advanced Bio Peptide Mapping C_18_ Column (2.1 × 150 mm, 2.7 µm) through ultra-low dispersion kit (5067–5963 Agilent Technologies), maintained at 60°C in thermostatted column compartment (TCC) and eluted at 0.4 mL/min flow rate with the following linear gradient: *t* (min), % B: 0, 2.5; 5,6; 64, 22; 85, 29; 90, 34; 95, 81; 97, 81; 97, 2.5; stop time, 99 min. The ESI source parameters operating in positive ion mode were as follows; gas temp., 300°C; drying gas, 6 L/min; nebulizer, 35 psi; sheath gas temp., 275°C, sheath gas flow, 12 L/min; *V*cap, 4500 V. The peptides were analyzed in the positive 4-bit multiplexing IM-QTOF mode in the *m/z* range of 290–1700 with a maximum drift time of 50 ms using nitrogen as drift gas, trap fill time of 3.2 ms; trap release time of 0.3 ms, and acquisition rate of 1 IM frame/s. The drift tube was operated with an absolute entrance voltage of 1700 V and an exit voltage of 250 V (drift field 18.529 V/cm) and the trapping funnel RF was set at 150 V. An Agilent ESI-Low Calibration mixture was injected both before the analysis to tune the instrument in the *m/z* range of 100–1700 and at the start of the worklist to perform single-field Collisional Cross Section (^DT^CCSN_2_) recalibration. The drift gas upgrade kit maintained both the drift tube and trap funnel pressure at constant 3.94 ± 0.01 and 3.80 ± 0.02 Torr, respectively, while the drift tube ambient temperature was stable at 23.5 ± 0.3°C across all the acquisition runs.

### LC-QQQ-MRM mass spectrometry

An Agilent 1200 Infinity series UHPLC system connected to 6495 QQQ (Agilent Technologies, USA) was used for the LC-ESI-QQQ-MRM assay. Mobile phase A consisted of 0.1% FA in water and mobile phase B of 0.1% FA in 100% ACN. Samples were loaded onto an Advanced Bio Peptide Mapping C_18_ Column (2.1 × 150 mm, 2.7 µm) maintained at 55°C in TCC and eluted at 0.4 mL/min flow rate with the following gradient, 2.5% B, 0 min; 6% B, 5 min; 9% B, 20 min; 22% B, 25 min; 29% B, 35 min; 34% B, 37 min; 81% B, 38 min; 81% B, 40 min; 2.5% B, 41 min with a post-run equilibration for 2 min. The list of transitions along with their retention times (RT) are presented in Supplementary Table 2. The source ESI parameters as well the collision energies were optimized for these peptides in the positive ion mode. The typical parameters were: gas temperature 200°C, gas flow 15 L/min, nebulizer 40 psi, sheath gas temperature 250°C and sheath gas flow 11 L/min. The capillary voltage was 4500 V and the nozzle voltage was set at 1000 V. The optimized iFunnel parameters were 150  and 60 V for high- and low-pressure RF, respectively. A total of 20 µL of LysN digested Na_2_CO_3_ SISCAPA samples were injected on to the columns.

### Data processing and statistical analyses

The IMS-MS data files collected using 4-bit multiplexing mode were first de-multiplexed using vendor-supplied software without any smoothing applied.^50^ Data post-processing, including ^DT^CCSN_2_ calibration and feature finding was carried out using IM-MS browser and Mass Profiler from MassHunter Suite (B.08.00, Agilent Technologies, Santa Clara, USA). Following post-processing, the raw data were imported into Skyline (v4.2) with formula annotations of the targeted peptides added to the method. Data for each peptide was extracted in the software in a MS1 filtering mode^51^ using the accurate mass of the top three isotopic peaks, drift time and RT for the precursor list workflow. The peak abundance for the Aβ_1__–__15_, Aβ_4__–__15_, Aβ_16__–__27_, Aβ_28__–__40_ and Aβ_28__–__42_ peptides in this study were performed on the first three peaks of the isotope cluster. The peak areas of the endogenous peptides and their heavy analogues (R = ^13^C_6_, ^15^N_4_, K = ^13^C_6_, ^15^N_2_) were extracted to derive the light-to-heavy ratios. The absolute quantification was determined by comparing the peak areas of the SIS peptides.

For the drift tube IMS, the resolving power *R* and resolution *r* are defined as *R* = *t*_d_/*w* and *r *=* *1.18 * (*t*_d1_ − *t*_d2_)/(*w*_1_ + *w*_2_), where *t*_d_ is the drift time of the ion and *w* is the full peak width at half-maximum (FWHM). To determine statistically significant differences between potential biomarkers (healthy versus Alzheimer’s disease brain tissue), the unpaired independent sample *t*-test was used, while Pearson’s correlation was used to assess correlation between different cell fractions. For the total amount of isomers and their normalized ratio, adjusted *P* values were calculated with one-way analysis of variance (ANOVA), corrected for multiple comparison false discovery rate (*P *<* *0.05) with Benjamini–Hochberg correction. The means of most common isomers of Aβ_1__–__15_ and Aβ_4__–__15_ were summarized as pie charts for Alzheimer’s disease and control brains, respectively, obtained from different biochemical fractions.

The degree of amyloid pathology was assessed in the *post-mortem* temporal cortex tissue using anti-Aβ immunohistochemistry (IHC) and semi-quantitatively scored by an independent assessor (CAM) after anti-Aβ (aa 8–17; 6 F/3D) staining. The scoring system comprised of; the following four categories: −, absent or not discernible, +, slight; ++, moderate; +++, severe. The semi-quantitative IHC scores were compared with the quantitative results obtained using IMS-MS in this study.

### Data availability

Patient and post-mortem brain tissue demographics, experimental details of nano-LC-ESI-MS/MS for ETD-PRM, MRM transition list for Aβ SISCAPA on QQQ, age-of-death correlation with absolute quantity of Aβ peptides and the respective isomers are provided in the supporting information. Additional data related to this article may be requested from the authors.

## Results

### Characterization of epimerization of Asp residues in brain-derived Aβ

Qualitative bottom-up proteomic identification of Aβ peptides in Alzheimer’s disease brain tissue demonstrated a range of N-terminal truncations including Aβ_1__–__15,_ Aβ_2__–__15_, Aβ_3Glu-15_, Aβ_4__–__15_ and the two canonical C-terminal peptides, Aβ_28__–__40_ and Aβ_28__–__42_ (Supplementary Table 3).^13^ Previous reports characterizing stereoisomers of synthetic Aβ have particularly demonstrated that isomerization at position Asp-1 and Asp-7 is frequent.^23^^,^^38^^,^^52^ However, neither the extent of isomerization of Asp-1 and Asp-7 residues in Aβ_1__–__15,_ Aβ_2__–__15_, Aβ_3Glu-15_ and Aβ_4__–__15_ have been directly measured in human brain nor has a systematic study been conducted using ion mobility to determine the effect of isomerization on the structure of these peptides in the gas phase. We postulated that the orthogonality of the online LC and drift tube ion mobility separations (DT-IMS) would provide the required analytical resolution, even if at modest *R* ∼ 50, to resolve the N-terminal Aβ isomers/racemers derived from Alzheimer’s disease brains and improve the detection/quantification limits of these complex biological samples. We applied DT-IMS in combination with chromatography and synthetic heavy labelled Aβ standards to characterize the identity of isomerized Aβ_1__–__15_, Aβ_2__–__15_, Aβ_3Glu-15_ and Aβ_4__–__15_ from Alzheimer’s disease brain.

**Figure 2:**
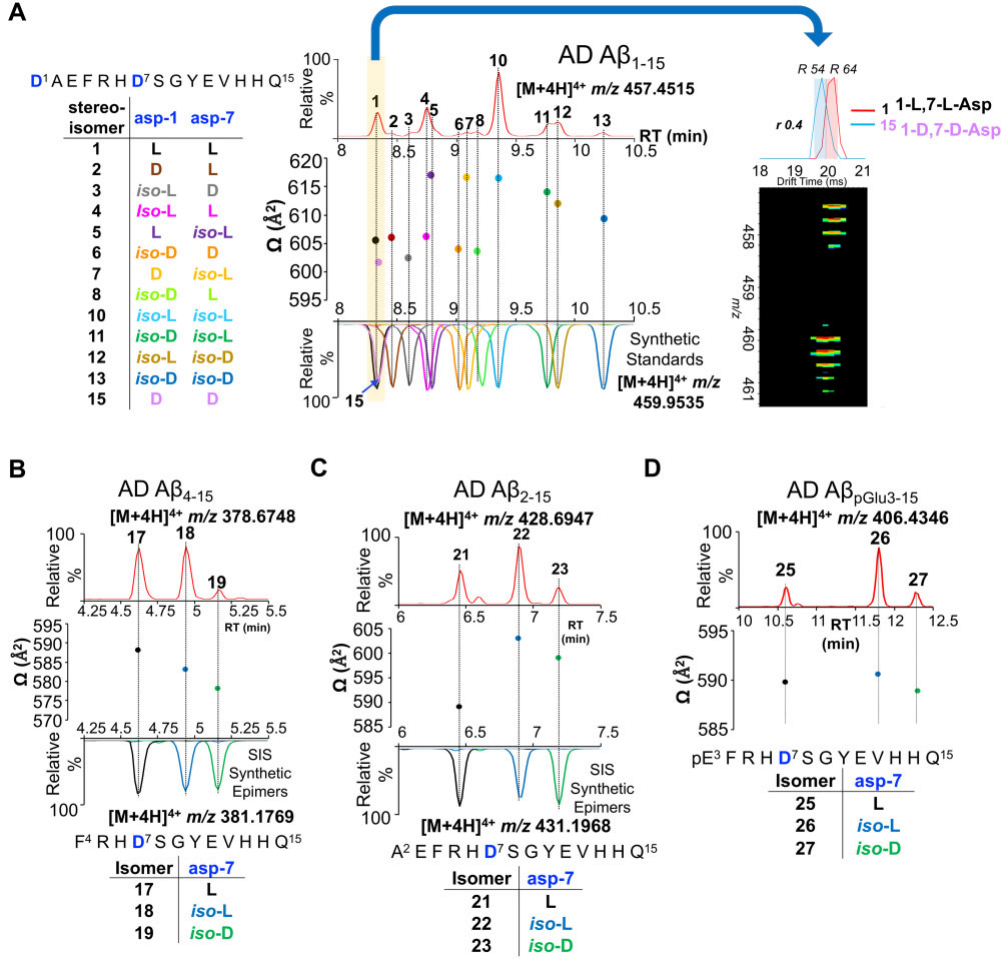
**Ion-mobility of amyloid-β isomers. Two-dimensional representation of RT_−_ion mobility high-resolution mass spectrometry (2D-LC-IMS-MS) results of extracted ion chromatograms (EIC) of N-terminal Aβ peptides present in Alzheimer’s disease brain.** (**A**) 2D-LC-IMS-MS of Aβ_1–15_ [M + 4H]^4+^*m/z* 457.4515 from formic acid fraction of human Alzheimer’s disease case illustrating the diversity of the isomerized Asp-1 and Asp-7 residues. The most abundant endogenous isomer of Aβ_1–15_ (*top red panel*) were characterized by comparing their chromatographic separation (co-elution) and their ^DT^CCSN_2_ (Å^2^) with the synthetic standards (*bottom multiple colour panel*). The alignment of both the LC as well as the ^DT^CCS_N2_ (Å^2^) reveal the most abundant endogenous isomers of Aβ_1–15_ in the FA fraction are 1,7-l-Asp (1), 1-*iso*-l, 7-l-Asp (4), 1-l, 7-*iso*-l-Asp (5), 1-*iso*-l, 7-*iso*-l-Asp (10), 1-*iso*-d, 7-*iso*-l-Asp (11), 1-*iso*-l, 7-*iso*-d-Asp (12) and 1-*iso*-d, 7-*iso*-d-Asp (13). The epimerized peptides 1-d, 7-l-Asp (2), 1-*iso*-l, 7-d-Asp (3), 1-*iso*-d, 7-d-Asp (6), 1-d, 7-*iso*-d-Asp (7) and 1-*iso*-d, 7-l-Asp (8) are minor constituents. The highlighted (yellow) LC-MS region depicts co-elution of native 1-l, 7-l-Asp (1) and 1-d, 7-d-Asp (15) at 8.3 min, although minute Δ^DT^CCS_N2_ ∼ 5 indicates that endogenous species corresponds to 1-l, 7-l-Asp (1) native Aβ_1–15_. (**B**) 2D-LC-IMS-MS representation of endogenous Aβ_4–15_ [M + 4H]^4+^*m/z* 378.6748 (top *red panel*) compared to isomerized synthetic standards (*bottom panel*), (**C**) Aβ_2–15_ [M + 4H]^4+^*m/z* 428.6947 (*top red panel*) compared to isomerized synthetic peptide standards (*bottom panel*) and (**D**) endogenous Aβ_pGlu3–15_ [M + 4H]^4+^*m/z* 406.4328 (*top LC panel*, red). ^DT^CCS_N2_ (Ω in Å^2^) are shown for the corresponding isomerized peptides for clarity.

As Aβ_1__–__15_ has two Asp residues (Asp-1 and Asp-7) that can undergo individual isomerization/epimerization events, each peptide having combination of their l/d and/or *iso*-l/*iso*-d forms resulting in total 16 Aβ_1__–__15_ isomers. Hence, we systematically characterized the liquid chromatography retention time (LC-RT) and ^DT^CCSN_2_ properties of possible synthetic SIS Aβ_1__–__15_ peptides (Supplementary Fig. 2). Next, probable combinations of different isomers were spiked into the proteolytically digested FA fraction extracted from an Alzheimer’s disease brain (Supplementary Fig. 3). The alignment of the drift times of the endogenous [M + 4H]^4+^*m/z* 457.4515 ion and the predicted SIS isomer [M + 4H]^4+^*m/z* 459.9535 ion in IMS-MS (Supplementary Fig. 3) along with the chromatographic elution (Supplementary Fig. 4) confirmed the identities of the endogenous Aβ_1__–__15_ isomer. Based on chromatographic RT and averaged ^DT^CCSN_2_ (Fig. 2A), we were able to characterize the most abundant seven isomers of Aβ_1__–__15_ found in the FA fraction of Alzheimer’s disease brain (with increasing RT) as 1-l, 7-l-Asp (1); 1-*iso*-l, 7-l-Asp (4); 1-l, 7-*iso*-l-Asp (5); 1-*iso*-l, 7-*iso*-l-Asp (10); 1-i*so*-d, 7-*iso*-l-Asp (11); 1-*iso*-l, 7-*iso*-d-Asp (12) and 1-*iso*-d, 7-*iso*-d-Asp (13). Minor singly enantiomerized Aβ_1__–__15_ epimers present in the Alzheimer’s disease FA fraction (with increasing RT) were 1-d, 7-l-Asp (2) and 1-*iso*-l, 7-d-Asp (3); 1-*iso*-d, 7-d-Asp (6), 1-d, 7-*iso*-l-Asp (7) and 1-*iso*-d, 7-l-Asp (8) (Fig. 2A). The ^DT^CCSN_2_ of [M + 4H]^4+^ ions for Aβ_1__–__15_ epimers indicated a trend in ion mobility from d-Asp < l-Asp < *iso*-d-Asp < *iso*-l-Asp. The ^DT^CCS_N2_ also indicated that the N-terminal Asp-1 epimerization does not significantly influence the gas phase structure (Fig. 2D) compared to the natural 1-l, 7-l-Asp Aβ_1__–__15_ (1) peptide. However, internal Asp-7 isomerization not only influences the RT of the isomerized species compared to the natural 1-l, 7-l-Asp (1) Aβ_1__–__15_ peptide, but importantly leads to a significant increase in ^DT^CCS_N2_ (Δ^DT^CCS_N2_ ∼ 10, Fig. 2A). This indicates a change in the gas phase conformation for the [M + 4H]^4+^ ion of the Asp-7 isomerized Aβ_1__–__15_ peptides in comparison with its native form.

On closer inspection, unmodified 1-l, 7-l-Asp (1) and 1-d, 7-d-Asp (15) epimers had similar RT (Fig. 2A, highlighted) but were slightly distinguishable in DT-IMS with ^DT^CCS_N2_ 606 Å^2^ (Fig. 2a, black) and 601 Å^2^ (Fig. 2A, violet), respectively. Although a DT resolution of *r* ∼ 0.4 (*R* ∼ 60) between 1-l, 7-l-Asp (1) (t_d_ = 20.2 ms) and 1-d, 7-d-Asp (15) (*t*_d_ = 19.7 ms) epimers (Fig. 2A, inset) is not sufficient for baseline resolution (*r* ∼1.5–2 is optimal for baseline separation), the presence of both of these species would have led to a DT peak broadening for the endogenous [M + 4H]^4+^*m/z* 457.4515 ion. However, DT-IMS results indicated doubly racemized endogenous Aβ_1__–__15_ (Fig. 2A, inset) is not present above the limit of detection.

Similarly, we characterized the isomers of other common N-truncated isoforms of Aβ derived from Alzheimer’s disease brains (Fig. 2B–D, Supplementary Fig. 5). In particular, 2D-LC-IMS-MS correlation of the endogenous Aβ_4__–__15_ [M + 4H]^4+^ peptide with its synthetic isomerized standards demonstrated that along with unmodified 7-l-Asp (17), 7-*iso*-l-Asp (18) and 7-*iso*-d-Asp (19) are the primary isomers of Aβ_4__–__15_ present in Alzheimer’s disease brain (Fig. 2B, Supplementary Fig. 5 A). For Aβ_2__–__15_ [M + 4H]^4+^, the most common isomers were 7-*iso*-l-Asp (22) and 7-*iso*-d-Asp (23) along with unmodified 7-l-Asp (21) (Fig. 2C, Supplementary Fig. 5B). Based on the RT and trend in ^DT^CCSN_2_ of the above-characterized epimers/isomers of the three N-terminal Aβ peptides (Aβ_1__–__15,_ Aβ_4__–__15_ and Aβ_2__–__15_), we predicted the major isomers of Aβ_pGlu3__–__15_ as 7-l-Asp (25), 7-*iso*-l-Asp (26) and 7-*iso*-d-Asp (27) with increasing RT (Fig. 2D, Supplementary Fig. 5 C). This was further confirmed by the diagnostic *iso*-Asp-7 ions generated via electron transfer dissociation-parallel reaction monitoring (ETD-PRM) (Supplementary Fig. 6 and Dataset 1).

Similar to Aβ_1__–__15_ isomers, internal Asp-7 isomerization of Aβ_2__–__15_ led to a larger ^DT^CCS_N2_ for the [M + 4H]^4+^*m/z* 428.6934 compared to unmodified 7-l-Asp (21) peptide. In contrast, the [M + 4H]^4+^*m/z* 378.6791 *iso*-Asp-7 Aβ_4__–__15_ peptide isomer was more compact (smaller ^DT^CCS_N2_) compared to its unmodified version (Fig. 2B). No change in ^DT^CCS_N2_ (Fig. 2D, Supplementary Fig. 5 C) for the [M + 4H]^4+^*m/z* 406.4329 *iso*-Asp-7 Aβ_pGlu3__–__15_ isomer was observed compared to its unmodified peptide. The structural reorganization inside the peptide backbone due to isomerization influences the shape and size of these peptides in the gas phase. N-truncation along with the loss of basic amino acid residues might further influence the charge distribution of these isomerized peptide ions that can lead to ^DT^CCS_N2_ alteration compared to unmodified Asp-l peptides.

We also investigated if the mid-region peptide Aβ_16__–__27_ derived from Alzheimer’s disease brain exhibited any conformational changes due to Asp-23 isomerization, as previously proposed.^9^ The presence of a single endogenous Aβ_16__–__27_ species from Alzheimer’s disease brain indicated no isomerization, verified by ETD-PRM (Supplementary Fig. 7 A) and 2D-LC-IMS-MS (^DT^CCS_N2_ ∼ 387 Å^2^) (Supplementary Fig. 7B). Similarly, no isomerization for the endogenous C-terminal peptides Aβ_28__–__40_ (^DT^CCS_N2_ ∼ 372 Å^2^) and Aβ_28__–__42_ (^DT^CCS_N2_ ∼ 406 Å^2^) were detected in Alzheimer’s disease brain (Supplementary Fig. 7B).

### Absolute quantitation of N-terminal Aβ peptides and estimation of Asp-1 and Asp-7 isomerization

**Figure 3:**
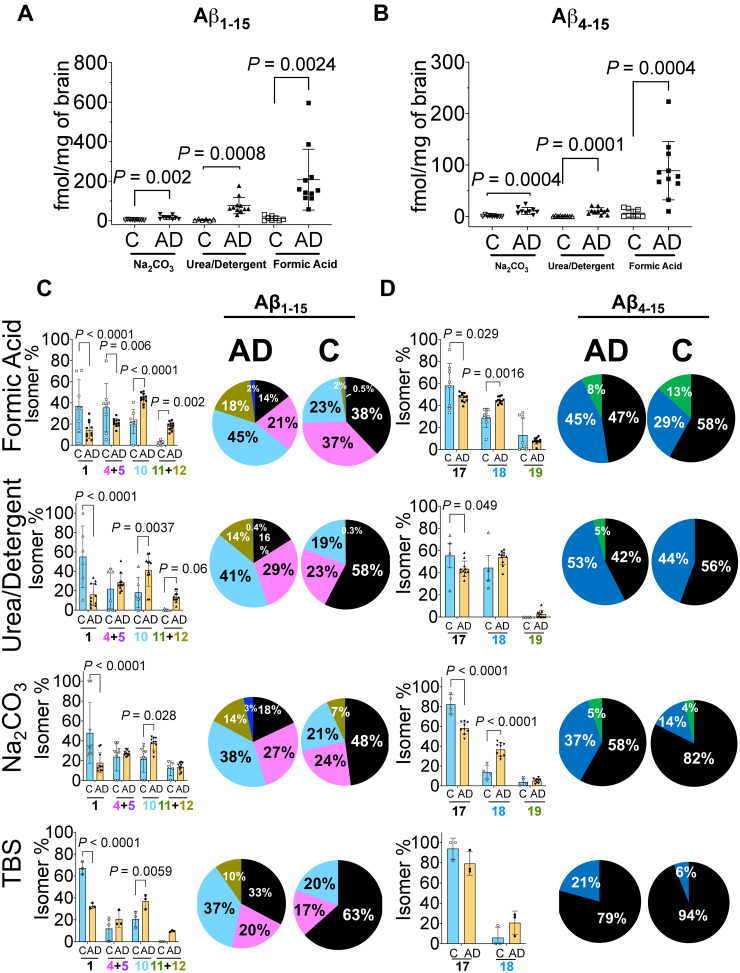
**Quantitation of amyloid-β N-terminus isomers. Scatter plots for the absolute quantitation of the N-terminus of Aβ peptides** (**A**) total Aβ_1–15_ and (**B**) total Aβ_4–15_ in the three amyloid rich biochemical fractions of Na_2_CO_3_, urea-detergent and formic acid. Aβ peptides were compared between Alzheimer’s disease (*n* = 11) and control (*n* = 9) using mass spectrometry. The total levels of Aβ_1–15_ and Aβ_4–15_ are significantly elevated in Alzheimer’s disease tissue in all biochemical fractions. (**C**) Percentage ratio of most abundant Aβ_1–15_ isomers and (**D**) Aβ_4–15_ isomers in Alzheimer’s disease compared to control brains across different biochemical fractions. The unmodified Aβ_1–15_ (native) is significantly decreased in all the biochemical fractions while doubly isomerized 1-*iso*, 7-*iso*-Asp Aβ_1–15_ (10) diastereomer is significantly increased. The Aβ_4–15_ isomer ratios demonstrated statistically significant changes in FA and Na_2_CO_3_ fractions. The pie charts summarize the pattern of distribution of isomerization of Aβ_1–15_ and Aβ_4–15_ in the different biochemical fractions. All the values are mean ± SD; significance in total Aβ_1–15_ and Aβ_4–15_ was determined by unpaired *t*-test with equal variance, while for the total amount of the Aβ_1–15_ and Aβ_4–15_ isomers and their normalized ratios, adjusted *p* values were calculated with ANOVA as described in the Materials and methods section. AD = Alzheimer’s disease; C = control, individual isomers of Aβ_1–15_ and Aβ_4–15_ are numbered according to Fig. 2.

Absolute quantitation of the Aβ_1__–__15_ and Aβ_4__–__15_ peptides in each of the four biochemical fractions of Alzheimer’s disease and control brain tissues was estimated using SIS peptides (Fig. 1C). As expected, total Aβ_1__–__15_ was significantly increased in Alzheimer’s disease brains (12-fold) compared to control brains, while 14-fold increase was documented for the total Aβ_4__–__15_ (Table 1). Comparison of Aβ_1__–__15_ and Aβ_4__–__15_ revealed a significant elevation in these peptides in Alzheimer’s disease across all four biochemical fractions (Table 1). Quantitative estimates of total Aβ_1__–__15_ in the FA fraction revealed a significant elevation (20-fold increase, *P *=* *0.0024), while 15-fold (*P *=* *0.0008) and 4-fold (*P *=* *0.0021) increase were observed in urea-detergent fraction and Na_2_CO_3_ fraction (Table 1, Fig. 3A), respectively. Due to low abundance of Aβ in the control group, we had to pool the TBS fractions for both the Alzheimer’s disease as well as controls cases. Pooled soluble TBS fraction indicated a 4-fold (*P *=* *0.0365) elevation of Aβ_1__–__15_ in Alzheimer’s disease brains (Table 1, Supplementary Fig. 8 A). Similarly, total Aβ_4__–__15_ was significantly increased (∼13-fold, *P *=* *0.0004) in FA fraction, ∼25-fold increase (*P *=* *0.0001) in urea-detergent fraction and ∼12-fold increase (*P *=* *0.0004) in Na_2_CO_3_ fraction (Table 1, Fig. 3B). Interestingly, we observed ∼29-fold increase (*P *=* *0.0263) in Aβ_4__–__15_ levels in the pooled Alzheimer’s disease TBS fraction (Supplementary Fig. 8 A). The Aβ_4__–__15_/Aβ_1__–__15_ ratio in the FA and Na_2_CO_3_ fractions was at ∼0.4 and 0.7, respectively, while this ratio in urea-detergent fraction was ∼0.1 (Table 1, Fig. 2A and B). This indicates preferential accumulation of Aβ_4__–__15_ in the insoluble/fibrillar and vesicular fractions in Alzheimer’s disease brains consistent with its increased hydrophobicity.^53^ The Aβ_4__–__15_/Aβ_1__–__15_ ratios in the insoluble pool indicated an increased accumulation of truncated Aβ with Phe-4 (phenylalanine residue) N-terminus in Alzheimer’s disease, reaching almost equal concentration as the BACE1 cleaved Aβ N-terminus (Asp-1), making it the next most abundant N-termini present in Alzheimer’s disease brains.

**Table 1:** Demographics and quantitation of Aβ from frontal cortex

	Alzheimer’s disease (*N *=* *11)	Control (*N = 9*)	*P*
Age (years)	84.19 (11.35)	69.90 (10)	0.01
PMI (h)	29.5 (20.2)	47.5 (22.8)	0.07
Formic acid (fmol/mg brain)			
Aβ_1__–__15_	207.8 (154.1)	11.43 (11.35)	0.0024
Aβ_4__–__15_	89.03 (56.68)	6.47 (7.67)	0.0004
Aβ_16__–__27_	4345 (1833)	368.0 (421.2)	<0.0001
Aβ_28__–__40_	326.1 (540.3)	5.63 (10.96)	0.1144
Aβ_28__–__42_	2278 (1090)	233.7 (303.0)	0.0003
Aβ_28__–__42_/Aβ_28__–__40_	44.75 (43.51)	97.47 (135.3)	0.2448
Urea-detergent (fmol/mg brain)			
Aβ_1__–__15_	76.63 (41.47)	4.10 (3.53)	0.0008
Aβ_4__–__15_	10.84 (6.32)	0.44 (0.66)	0.0001
Aβ_16__–__27_	817.9 (732.1)	198.5 (204.4)	0.036
Aβ_28__–__40_	24.44 (57.69)	1.69 (4.27)	0.2848
Aβ_28__–__42_	906.0 (692.8)	242.4 (255.8)	0.0207
Aβ28_–__42_/Aβ_28__–__40_	1241 (2364)	2368 (3396)	0.3936
Na_2_CO_3_ (fmol/mg brain)			
Aβ_1__–__15_	16.16 (9.78)	4.0 1(1.72)	0.0021
Aβ_4__–__15_	10.91 (6.7)	0.86 (1.32)	0.0004
Aβ_16__–__27_	118.0 (76.73)	25.0 (20.30)	0.0047
Aβ_28__–__40_	5.92 (7.89)	2.48 (0.51)	0.2102
Aβ_28__–__42_	33.61 (19.87)	9.39 (7.28)	0.0001
Aβ_28__–__42_/Aβ_28__–__40_	8.02 (4.08)	3.96 (3.16)	0.0311
TBS (fmol/mg brain)	**Alzheimer’s disease** (***N = *9**)	Control (***N = *9**)	
Aβ_1__–__15_	16.4 (0.9)	4.92 (1.0)	0.0365
Aβ_4__–__15_	2.01 (0.79)	0.07 (0.03)	0.0263
Aβ_16__–__27_	22.28 (4.93)	7.34 (2.3)	0.0093
Aβ_28__–__40_	1.23 (0.54)	0.56 (0.47)	0.2572
Aβ_28__–__42_	0.18 (0.2)	0.05 (0.01)	0.3960
Aβ_28__–__42_/Aβ_28__–__40_	0.29 (0.46)	0.18 (0.16)	0.7225
Total (fmol/mg brain)	**Alzheimer’s disease (*N *=* *11)**	**Control (*N = *9)**	
Aβ_1__–__15_	297.7 (193.5)	16.9 (15.73)	0.0009
Aβ_4__–__15_	108.8 (66.42)	7.78 (9.28)	0.0008
Aβ_16__–__27_	5260 (2190)	525.8 (614)	<0.0001
Aβ_28__–__40_	355.4 (599.6)	8.99 (14.95)	0.0844
Aβ_28__–__42_	3211 (1156)	406.6 (502.9)	<0.0001

All values are mean values (±SD); significance was determined by unpaired *t*-test with equal variance.

Next, we asked how much of total Aβ_1__–__15_ and Aβ_4__–__15_ was isomerized in each biochemical fraction in Alzheimer’s disease in comparison to the control tissue. As expected, we observed significant elevation in the total amount of the isomers of Aβ_1__–__15_ and isomers of Aβ_4__–__15_ in each of the Aβ rich fractions from Alzheimer’s disease tissue (Supplementary Fig. 9). Interestingly, even in the pooled Alzheimer’s disease TBS fraction (soluble Aβ), we not only observed a significant decrease in the unmodified 1-l, 7-l-Asp Aβ_1__–__15_ (1) peptide, but statistically significant elevation for the singly isomerized 1-*iso*-l, 7-l-Asp (4), 1-l, 7-*iso*-l-Asp (5) and doubly isomerized 1,7-*iso*-l-Asp Aβ_1__–__15_ (10) peptides (Supplementary Fig. 8B).

In order to understand how the isomerization of Asp-1 and Asp-7 was associated with Alzheimer’s disease, we investigated the changes in the total percentage of each isomer/epimer across the biochemical fractions (Fig. 3C and D). The percentage of isomerized to unmodified Aβ_1__–__15_ indicated significant decrease of the native 1-l, 7-l-Asp (1) peptide with the concomitant statistically significant increase in 1-*iso*-l-Asp, 7-*iso*-l-Asp Aβ_1__–__15_ (10) isomer in all the biochemical fractions in Alzheimer’s disease (Fig. 3C). Overall, ∼85% of Aβ_1__–__15_ was detected in its isomerized form in the amyloid-rich fractions of Alzheimer’s disease, while controls showed up to 50% isomerization depending on the pathology (Fig. 3C, Supplementary Fig. 10). Quantitatively, ∼50% isomer 10 in the most amyloid-rich fractions of Alzheimer’s disease brains compared to 20–27% in controls (Fig. 3C) was documented. Furthermore, we observed ∼21–30% Aβ_1__–__15_ with either Asp-1 or Asp-7 isomerized in Alzheimer’s disease. In contrast, in controls singly isomerized Asp-1 or Asp-7 Aβ_1__–__15_ are the predominant species in the FA fractions (∼37%, *P *=* *0.006) (Fig. 3C). These data indicated an increased isomerization event of Aβ_1__–__15_ in Alzheimer’s disease brain for an extended period of time. Strikingly, even the TBS soluble Aβ_1__–__15_ present in the Alzheimer’s disease brains demonstrated ∼70% isomerization (∼37% isomer 10, *P *=* *0.0059) compared to only ∼30% in controls (Fig. 3C).

Similarly, the proportion of isomerized Aβ_4__–__15_ was elevated in Alzheimer’s disease brain (Supplementary Fig. 8B). There was a significant increase of 7-*iso*-l-Asp Aβ_4__–__15_ (18 in Fig. 3D) in FA (45%, *P *=* *0.0016) and Na_2_CO_3_ (37%, *P *<* *0.0001) fractions. Most importantly, there was an increase (∼21%) in the amount of 7-*iso*-l-Asp Aβ_4__–__15_ (18 in Fig. 3D) in the soluble TBS fractions of Alzheimer’s disease brains compared to control brains (Fig. 3D). We also observed a distinct pattern of compartmentalization of highly isomerized Aβ in the insoluble fractions, while the soluble TBS pool had a higher percentage of unmodified peptide (Fig. 3C and D).

The potential influence of age at death on the accumulation of isomerized Aβ in the Alzheimer’s disease brains was evaluated. As expected, the total levels of Aβ_1__–__15_ and Aβ_4__–__15_ were positively correlated with the age at death in the Alzheimer’s disease brains (Supplementary Fig. 11) but not in control brains (Supplementary Fig. 12). However, no correlations with the isomer ratios of Aβ_1__–__15_ or Aβ_4__–__15_ were observed in Alzheimer’s disease or controls (Supplementary Figs. 11 and 12). These results corroborate the spontaneous non-enzymatic reaction as the primary mechanism for the generation of these isomers on long-lived Aβ in the brains (not artefact of sample preparation).

### Aβ mid-domain and C-terminus quantitation

**Figure 4:**
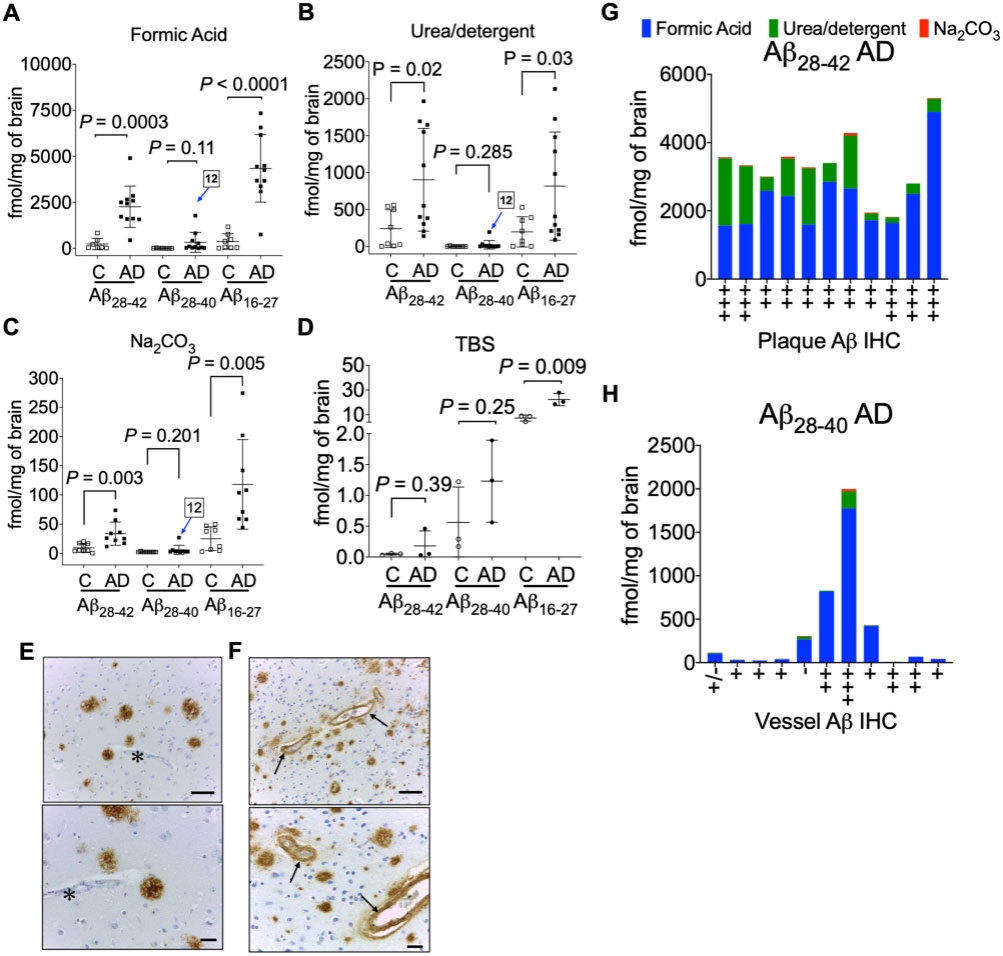
**Quantitation of amyloid-β C-terminus. Scatter plots for the absolute quantitation of C-terminal Aβ_28–42_, Aβ_28–40_ and mid-domain Aβ_16–27_ in the** (**A**) FA, (**B**) urea-detergent, (**C**) Na_2_CO_3_ and (**D**) soluble TBS fractions from 9 controls and 11 Alzheimer’s disease brains (temporal cortex). Pooled TBS homogenates (three replicates of pooled control and pooled Alzheimer’s disease) were used to estimate the Aβ_28–42_, Aβ_28–40_ and mid-domain Aβ_16–27_ levels. The levels of Aβ_16–27_ were significantly elevated in Alzheimer’s disease in all the biochemical fractions, while Aβ_28–42_ was significantly elevated in FA, urea-detergent and Na_2_CO_3_ fractions. No statistical alteration was found in the levels of Aβ_28–40_ in Alzheimer’s disease compared to controls. AD12 indicates the patient with a high Aβ_28–40_ level compared to others in all the amyloid-enriched biochemical fractions. Representative immunohistochemistry (IHC) images demonstrating Aβ amyloid staining in (**E**) typical Alzheimer’s disease plaques without any vascular amyloid (asterisk) and (**F**) plaques and the intima of small blood vessels (arrows) from patient AD12 with the unusually high Aβ_28–40_ level. Scale bar = 100 µm. (**G**) Total Aβ_28–42_ levels compared to the amyloid plaque burden and (H) total Aβ_28–40_ levels compared to vessel amyloid quantification from IHC. All the values are mean ± SD; significance in total Aβ_28–42_, Aβ_28–40_ and Aβ_16–27_ was determined by unpaired *t*-test with equal variance. AD = Alzheimer’s disease; C = control.

We hypothesized the Aβ_28__–__42_ and Aβ_28__–__40_ quantitation would provide information about the distribution of the most common type of Aβ accumulating in the Alzheimer’s disease brain tissue as it is generally assumed Aβ_42_ is the predominate neuronal form of the peptide.^18^^,^^54^ The absolute quantitation of Aβ_28__–__42_ and Aβ_28__–__40_ peptides was used to determine the ratio of Aβ_40_/Aβ_42_ peptides across all the biochemical fractions (Supplementary Fig. 13). The quantitative estimates of Aβ_28__–__42_ in the FA fraction was 2259 ± 1123 fmol/mg of brain in Alzheimer’s disease tissue versus 233.7 ± 303 fmol/mg (Fig. 4A) in control brain (∼10-fold increase *P *=* *0.0003). In the urea-detergent fraction, we documented only a 3-fold increase (*P *=* *0.0207) of Aβ_28__–__42_ (Table 1, Fig. 4B) in Alzheimer’s disease brain (903.9 ± 695.3 fmol/mg brain) compared to control brain (324.4 ± 255.8 fmol/mg brain). The Aβ_28__–__42_ SISCAPA quantitation in the Na_2_CO_3_ fraction indicated a ∼3.5-fold increase (*P = *0.0001) (Table 1, Fig. 4C) in Alzheimer’s disease brain compared to control brain. Total Aβ_28__–__42_ in the controls with pathology exhibited a range from 400 to 1400 fmol/mg brain (Supplementary Fig. 14) compared to 400 fmol/mg in non-pathological controls. Most interestingly, although there was more Aβ_28__–__40_ in Alzheimer’s disease compared to control tissue, it did not reach statistical significance in all of the biochemical fractions (Table 1). Strong correlation between Aβ_28__–__42_ and Aβ_16__–__27_ levels was found in the amyloid-rich biochemical fractions (Supplementary Fig. 15), while Aβ_28__–__40_ did not correlate with Aβ_16__–__27_ levels (Supplementary Fig. 16). This was driven by the high levels of Aβ_28__–__42_, the predominant C-terminal peptide that accumulates in sporadic Alzheimer’s disease brains.

As the decrease in the ratio of Aβ_42_/Aβ_40_ in the biological fluids (CSF/blood) has been shown to inversely correlate with brain amyloid PET imaging,^55^^,^^56^ we next compared the ratio of Aβ_28__–__42_ and Aβ_28__–__40_ (Table 1, Supplementary Fig. 13). We did not find a corresponding increase in the Aβ_42_/Aβ_40_ ratio in FA or urea-detergent fractions but observed a statistically significant increase in Aβ_42_/Aβ_40_ ratio in the Na_2_CO_3_ fraction in Alzheimer’s disease brains (Supplementary Fig. 13). This suggests the mechanisms that decrease Aβ_42_ levels in CSF are disconnected with corresponding peptide levels in the brain. During our analysis, we observed an individual with highly elevated Aβ_28__–__40_ in one Alzheimer’s disease brain tissue (case 12, Fig. 4A–C). The high level of Aβ_28__–__40_ correlated with the presence of prominent perivascular Aβ-amyloidosis for this case (Fig. 4F), as has been previously reported.^54^^,^^57^ While the total Aβ_28__–__42_ (3300 ± 974 fmol/mg brain) was consistent in Alzheimer’s disease patients (Fig. 4G), significant increase in Aβ_28__–__40_ was observed with prominent perivascular Aβ-amyloidosis (Fig. 4H).

## Discussion

The slowly progressive nature of Alzheimer’s disease with almost 20 years of Aβ accumulation from threshold to the onset of dementia predisposes the depositing Aβ peptide to undergo multiple biochemical changes at the molecular level. We used a quantitative proteomics approach coupled with ion mobility mass spectrometry to unravel the diversity of isomerized Aβ N-termini found in the Alzheimer’s disease brain. Our major findings include (i) characterization of isomerization of the Asp residues (Asp-1 and Asp-7) in four common sequentially truncated N-termini of Aβ found in Alzheimer’s disease brain tissues; (ii) quantitative estimation of the level of Aβ_1__–__15_ and Aβ_4__–__15_ in the biochemical pools with significant elevation in the Alzheimer’s disease brain tissue; (iii) evaluation of the isomer ratios of Aβ_1__–__15_ and Aβ_4__–__15_, with significant elevation in doubly isomerized Aβ_1__–__15_ and isomerized Aβ_4__–__15_ levels in the insoluble/fibrillar and membrane pools, while the sparsely populated vesicular and soluble Aβ pools have lower proportion of these PTMs; (iv) brain-derived Aβ primarily has Ala-42 as the C-terminus which is significantly increased in Alzheimer’s disease, while Aβ with Val-40 C-terminus is increased in Alzheimer’s disease but does not reach statistical significance compared to control brains.

Iso-aspartate formation is one of the most common modifications associated with long-lived proteins/peptides.^22^^,^^58^ The rate of *iso*-Asp formation in model peptides is considerably slower (half-life *t*_1/2_, 53–266 days depending on the sequence) compared to asparagine deamidation/isomerization (*t*_1/2_, 1.4 days). *In vitro*, the N-terminus of Aβ has been documented to undergo such spontaneous isomerization at Asp1 and Asp7 residues.^23^ Slow reaction rates (*t*_1/2_ ∼ 231 days, Asp-1; *t*_1/2_ ∼ 462 days, Asp-7 for Aβ_1__–__40_)^12^ along with decreased fractional clearance rates in the CNS of Alzheimer’s disease (28 ng/h Aβ_1__–__42_ deposition)^5^^,^^59^ can be used to estimate the age of the depositing isomerized peptides. Pathological controls with ∼50% isomerized Aβ_1__–__15_ (one *t*_1/2_) indicate that the observed mild Aβ deposit (diffuse plaques) is nearly 8 months old. In contrast, data from the ∼85% isomerized Aβ_1__–__15_ (three *t*_1/2_) in Alzheimer’s disease (Fig. 3C), indicate that the age of this peptide is at least 4 years. Similarly, the age of Aβ_4__–__15_ in Alzheimer’s disease is nearly 1.2 years compared to 6 months in control brains. Interestingly, the rate of racemization of l-Asp to d-Asp was originally used to estimate 30 years for plaques formation.^60^ Further investigation using better modelling of Aβ biogenesis and altered clearance rates observed in Alzheimer’s disease patients would yield better estimates for these long-lived PTMs.

Along with N-terminal Asp isomerization, sequential truncated isoforms of Aβ such as Aβ_pGlu_ have been well documented from different biochemical pools of Alzheimer’s disease brain.^61^ All of these PTMs have been linked to the hypothesis of how Aβ is toxic to neurons. However, they do not completely address the underlying feature of how or what causes the accumulation of Aβ to occur. Structural reorganization of the peptide chain due to Asp isomerization leads to alteration in the biochemical and physical properties of the peptide. Our data indicate that internal Asp residue isomerization reorients the peptide backbone, leading to changes in the shape and size of these Aβ peptides (Fig. 2) compared to unmodified ones in the gas phase. One of the possible links between the more stable long-lived isomerized/epimerized Aβ^62^ and neurotoxicity could stem from their inherent resistance to enzymatic degradation by primary cathepsin found in the lysosomes.^12^ Aβ residues 1–11 are predicted to play a critical role in the antigen recognition by antibodies targeting the N-terminus of Aβ peptide.^63–65^ It has been suggested that N-terminus of Aβ is the dominant epitope, exposed on the surface of aggregated fibrillary deposits, while Aβ mid-domain drives oligomerization and toxicity.^66^ Antibodies that target N-terminus are considered competent in reducing Aβ deposits, while antibodies to mid-domain epitopes^67^^,^^68^ should abrogate the toxic oligomers. Despite considerable reduction in Aβ (lowering of Aβ-PET signal) by monoclonal antibodies primarily to the Aβ N-terminus,^33^^,^^34^^,^^69^ active and passive immunotherapy trials have largely failed to reach their primary end points.^70^ Our data suggest, antibodies targeting the mid-domain Aβ might prove efficacious as it has very little PTM, while specifically targeting the older isomerized Aβ N-terminus for clearance will be better strategy for immunotherapy. Designing better therapeutic antibodies against modified Aβ would need further investigation into the structural properties of these PTMs and their influence on the antibody-mediated target engagement.

It has been postulated that the hydrophobic C-terminus of Aβ is responsible for inducing membrane permeability,^71^ while the N-terminal domain induces innate immune responses from the microglia. Interestingly, it has been found that *iso*-Asp-7 Aβ_42_ compared to wild-type Aβ_42_ led to significantly increased phosphorylation of proteins, including tau (MAPT) from SH-SY5Y neuroblastoma cell-culture models.^72^ Accumulation of iso-aspartate in proteins is known to be lethal in the PIMT (protein iso-aspartate methyltransferase) deficient mouse, suffering from progressive epileptic seizures.^73^^,^^74^ Soluble Aβ oligomers isolated from Alzheimer’s disease brains have been shown to induce hyperexcitability in individual neurons and neuronal circuits^75–77^ Induction of hyperexcitability has been invoked to explain the clinical observation that there is a significantly higher incidence of epilepsy in Alzheimer’s disease patients compared to age-matched controls.^78^^,^^79^ Our results indicate that soluble Aβ_1__–__15_ derived from Alzheimer’s disease brain is significantly isomerized (∼50% doubly isomerized, 20% singly isomerized) compared to soluble Aβ_1__–__15_ (∼20% doubly isomerized, 17% singly isomerized) in age-matched control brains (Fig. 3C). It would be interesting to quantitatively estimate how much of these Alzheimer’s disease brain-derived soluble Aβ oligomers are isomerized at the N-terminus.

While we documented abundant N-terminal Asp-1 and Asp-7 isomerization/racemization in all the four different biochemical pools in both Alzheimer’s disease and control brains (Fig. 3, Supplementary Fig. 10), surprisingly no modified Aβ_16__–__27_ was observed (Supplementary Fig. 7). The presence of unique Aβ_16__–__27_ species points to two major revelations: (i) N-terminus of Aβ is conformationally flexible allowing spontaneous reactions to occur and (ii) contrary to previous reports,^9^^,^^80^ Asp-23 is unmodified in sporadic Alzheimer’s disease. This indicates that this residue could either be solvent in-accessible or involved in H-bonding interactions precluding it from succinimide-mediated isomerization. With the current resolution of *R* ∼ 50 for our DT-IMS-MS method, it is not possible to rule out any other amino acid (such as Ser) isomerization on this peptide. Future investigations with techniques like SLIM-IMS providing higher resolution (*R *>* *300)^46^^,^^47^ will lead to better understanding and characterization of other low abundant structural PTMs of Aβ in Alzheimer’s disease brains.

The data presented here and by others^13^^,^^59^^,^^81–83^ is consistent with Aβ_42_ being the dominant neuronal peptide form accumulating in Alzheimer’s disease brain with Aβ_40_ levels increasing with perivascular amyloidosis.^54^ Label-free intact MS has estimated that ∼70% of Aβ depositing in the Alzheimer’s disease brain has Ala-42 as the C-terminus compared to ∼10% terminating at Val-40.^13^ Historically, the majority of the peptide originally sequenced from the plaque-derived amyloid was Aβ_4__–__42_.^7^ Our results indicate that Aβ peptides depositing specifically in the insoluble pools of Alzheimer’s disease brain have approximately equal amounts of BACE-1 cleaved Aβ (Asp-1 as the N-terminus) and ragged N-terminus peptide (Phe-4 residue) (Table 1, Fig. 3). Interestingly, recent MALDI-MS imaging of post-mortem Alzheimer’s disease tissues with congophilic amyloid angiopathy (CAA) provided a distinct qualitative pattern of N- and C-terminal variations of deposited Aβ—extracellular plaques in the cerebral parenchyma were enriched with Aβ_42_ while the vessels had less aggregation prone Aβ_40_.^83–85^ Quantitative estimation of Aβ_42_ and Aβ_40_ in biochemically defined pools from the temporal cortex of sporadic Alzheimer’s disease revealed less than 0.01% of total Aβ_42_ and 0.3% of total Aβ_40_ are in the soluble cytosolic TBS fraction with the rest being distributed in either the vesicular (1.1% Aβ_42_, 1.7% Aβ_40_), membranous (28.3% Aβ_42_, 6.8% Aβ_40_) and/or insoluble fully polymerized fibrillar phase (70.7% Aβ_42_, 91.2% Aβ_40_). In contrast, total Aβ_42_ in control brain tissue (0.5 ± 1.8 pmol/mg brain) is much lower concentrated compared to Alzheimer’s disease brain (3.2 ± 1.8 pmol/mg brain). Again, most of the Aβ_42_ is still partitioned in the membranous (57.2%) and insoluble/fibrillar fraction (41.2%). Drugs that can target the C-terminus,^86^ specifically Aβ_42_ for clearance have much better chance to exploit the equilibrium of amyloid deposition in Alzheimer’s disease brain.

Development of therapeutic drugs and interventions for ameliorating or decreasing the progress of Alzheimer’s disease requires techniques that can accurately and quantitatively monitor the changes in the amyloid biomarkers in the CSF and/or the blood along with PET imaging. Our results show that isomerized Aβ is intricately associated with the accumulation of Aβ_42_ in the brain_**—**_a key distinguishing signature from freshly generated Aβ. Future studies will be required to understand the role of these isomers in the disease, but this is clearly an important question to answer due to the >80% abundance of isomerized Aβ in Alzheimer’s disease brain.

## Conclusion

In summary, in this study we have shown that different biochemical pools of Aβ has different amounts of N-terminus isomerization. Insoluble plaques and membrane fractions in Alzheimer’s disease brains have ∼85% isomerized Aβ_1__–__15_, while vesicular and soluble fractions have lower percentage of isomerization. The extent of isomerization on Aβ extracted from Alzheimer’s disease brains is 3 years older than the Aβ found in age-matched control brains. Quantitatively, BACE-1-cleaved Asp-1 N-terminus is present in almost equimolar amounts with Phe-4 truncated N-terminus, an interesting Aβ metabolic by-product of unclear origin. Our data provide the link between older isomerized Aβ and the consequences it might have in the disease aetiology, such as oligomers that diffuse out of these plaques into soluble pool will be neurotoxic due to their inherent resistance to lysosomal degradation. Strategies in designing better immunotherapeutic must take into consideration of the extensive PTMs of the N-terminus of Aβ and specifically target older isomerized Aβ species for better target engagement and clearance.

## Supplementary material

Supplementary material is available at *Brain Communications* online.

## Supplementary Material

fcab028_Supplementary_Data

## Abbreviations

Aβ =: amyloid-β
Asp/Asn =: aspartate/asparagine
C =: control
CCS =: drift tube collisional cross-section
ESI =: electrospray ionization
ETD =: electron transfer dissociation
FA =: formic acid
IHC =: immunohistochemistry
IMS =: ion mobility separations
*m/z* =: mass-to-charge ratio
MS =: mass spectrometry
PET =: positron emission tomography
PRM =: parallel reaction monitoring
PTMs =: post-translational modifications
SIS =: stable isotope standard
SISCAPA =: stable isotope standards and capture by anti-peptide antibodies
TBS =: tris-buffered saline
UHPLC =: ultra-high-performance liquid chromatography
