# Supplementary material for: Quantification of N-terminal amyloid-β isoforms reveals isomers are the most abundant form of the amyloid-β peptide in sporadic Alzheimer’s disease
Source: Brain Commun. 2021 Mar 9;3(2):fcab028. doi: 10.1093/braincomms/fcab028 (PMC8062259; doi:10.1093/braincomms/fcab028)
Supplement: fcab028_Supplementary_Data [file fcab028_supplementary_data.zip › Supplemental_Dataset_1.pdf]

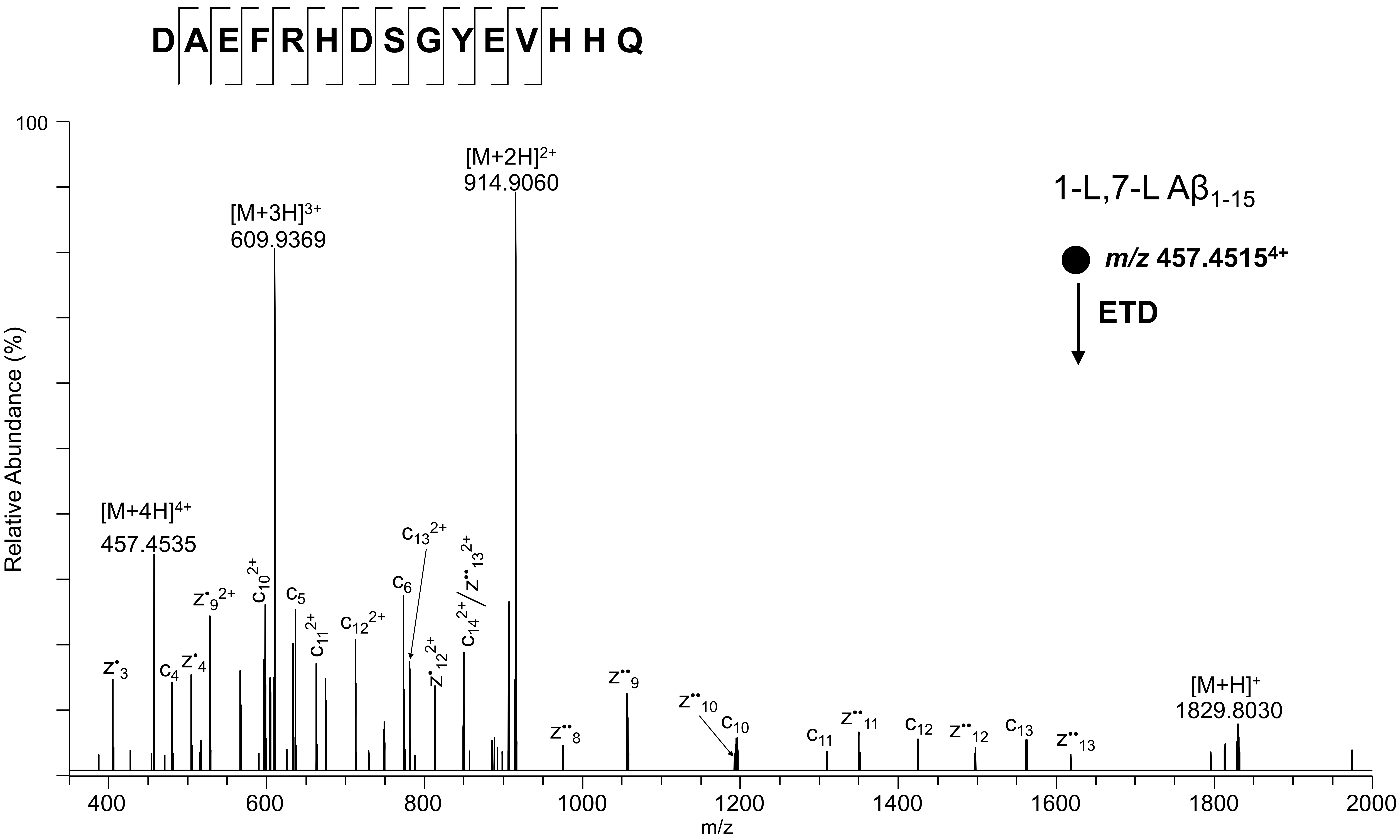
D A E F R H D S G Y E V H H Q
1-L,7-L Aβ1-15
m/z 457.4515 4+
ETD
[M+2H]2+
914.9060
[M+3H]3+
609.9369
[M+4H]4+
457.4535
[M+H]+
1829.8030
z•3
c4
z•4
z•9 2+
c10 2+
c5
c11 2+
c12 2+
c6
c13 2+
z•12 2+
c14 2+/z••13 2+
z••8
z••9
z••10
c10
c11
z••11
c12
z••12
c13
z••13
Relative Abundance (%)
100
m/z
400
600
800
1000
1200
1400
1600
1800
2000

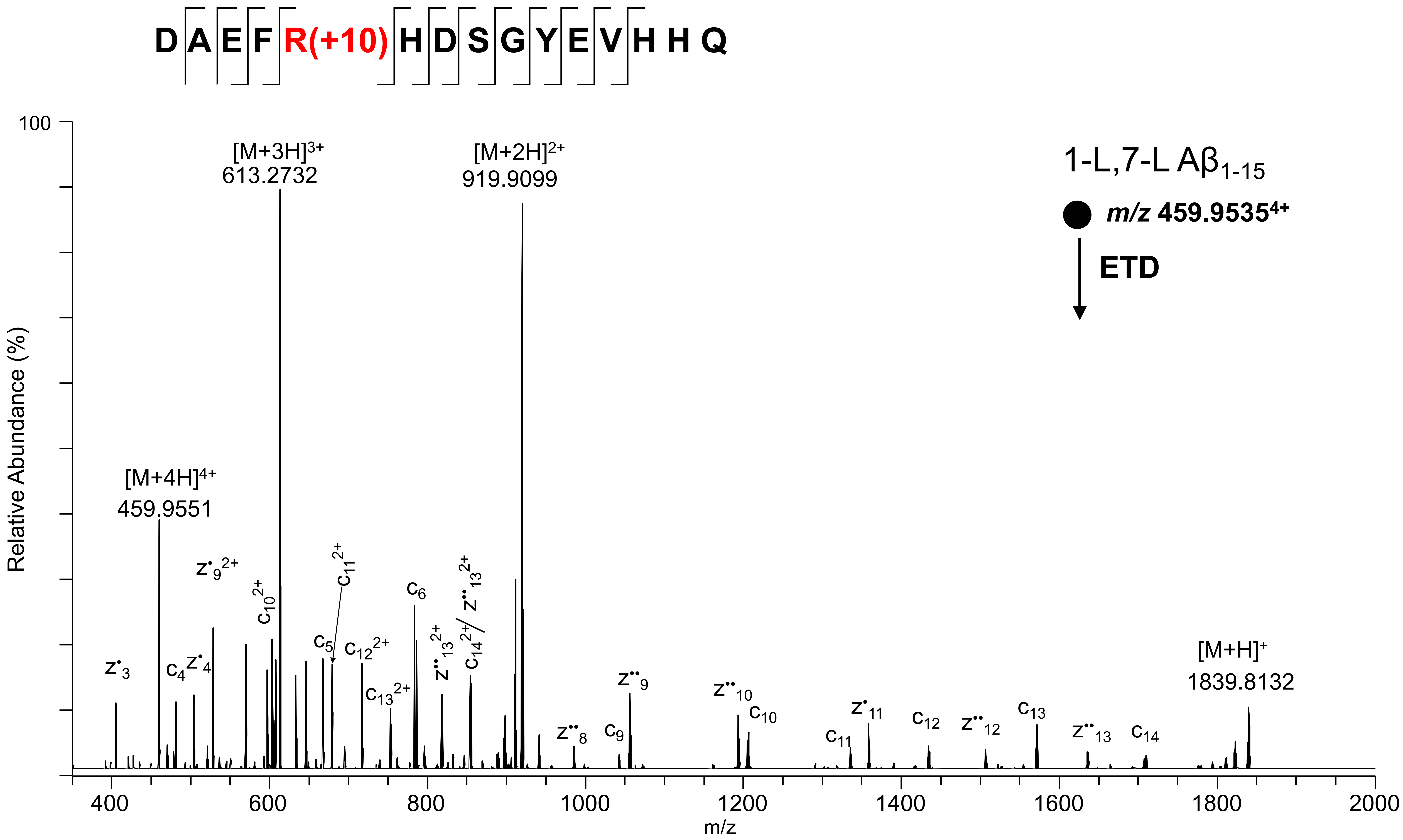

D A E F R(+10) H D S G Y E V H H Q
1-L,7-L Aβ1-15
m/z 459.9535⁴⁺
ETD
[M+3H]3+ 613.2732
[M+2H]2+ 919.9099
[M+4H]4+ 459.9551
[M+H]+ 1839.8132
Relative Abundance (%)
m/z
100
400
600
800
1000
1200
1400
1600
1800
2000

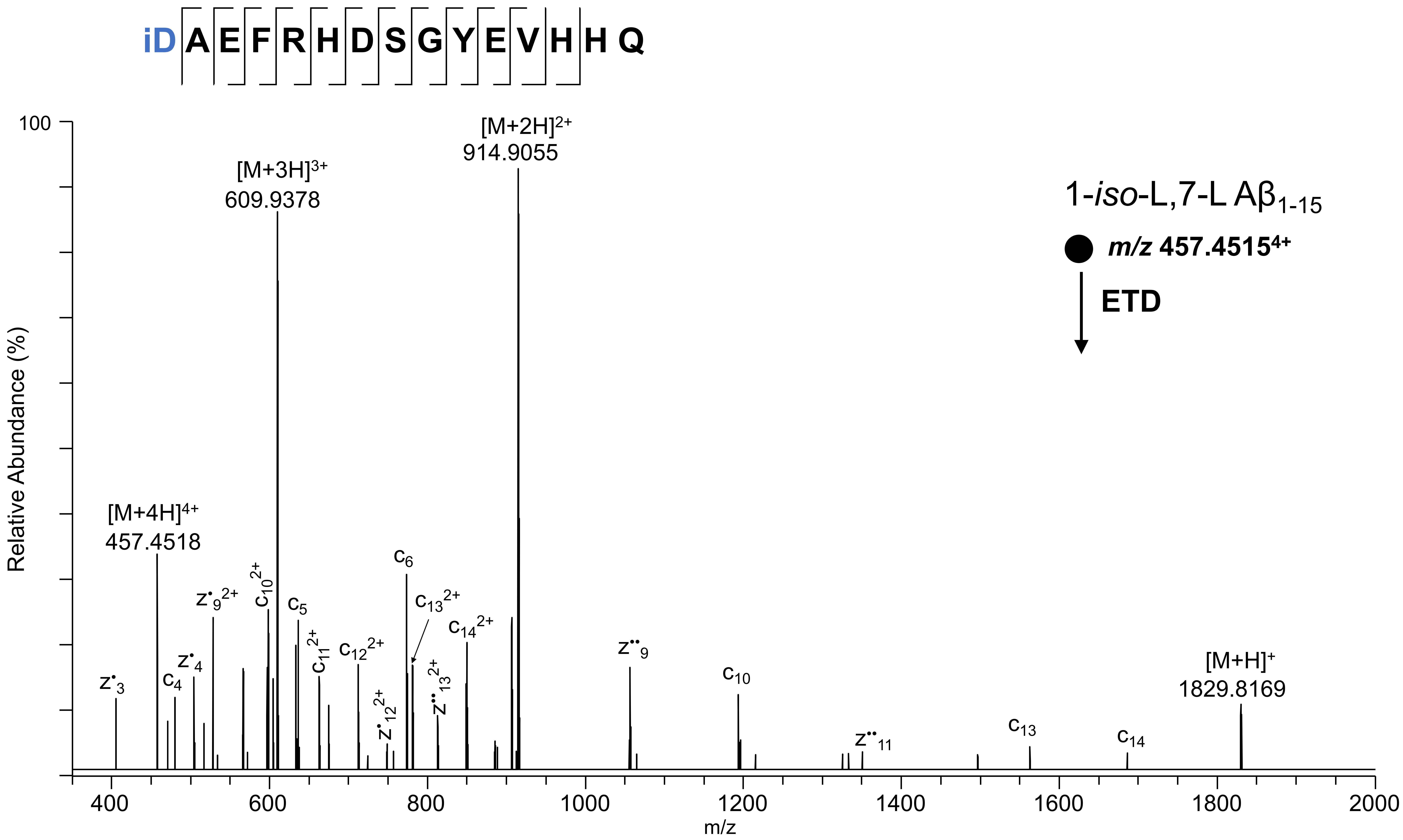

iD A E F R H D S G Y E V H H Q
1-iso-L,7-L Aβ1-15
m/z 457.4515 4+
ETD
[M+2H]2+
914.9055
[M+3H]3+
609.9378
[M+4H]4+
457.4518
[M+H]+
1829.8169
Relative Abundance (%)
m/z
100
400
600
800
1000
1200
1400
1600
1800
2000

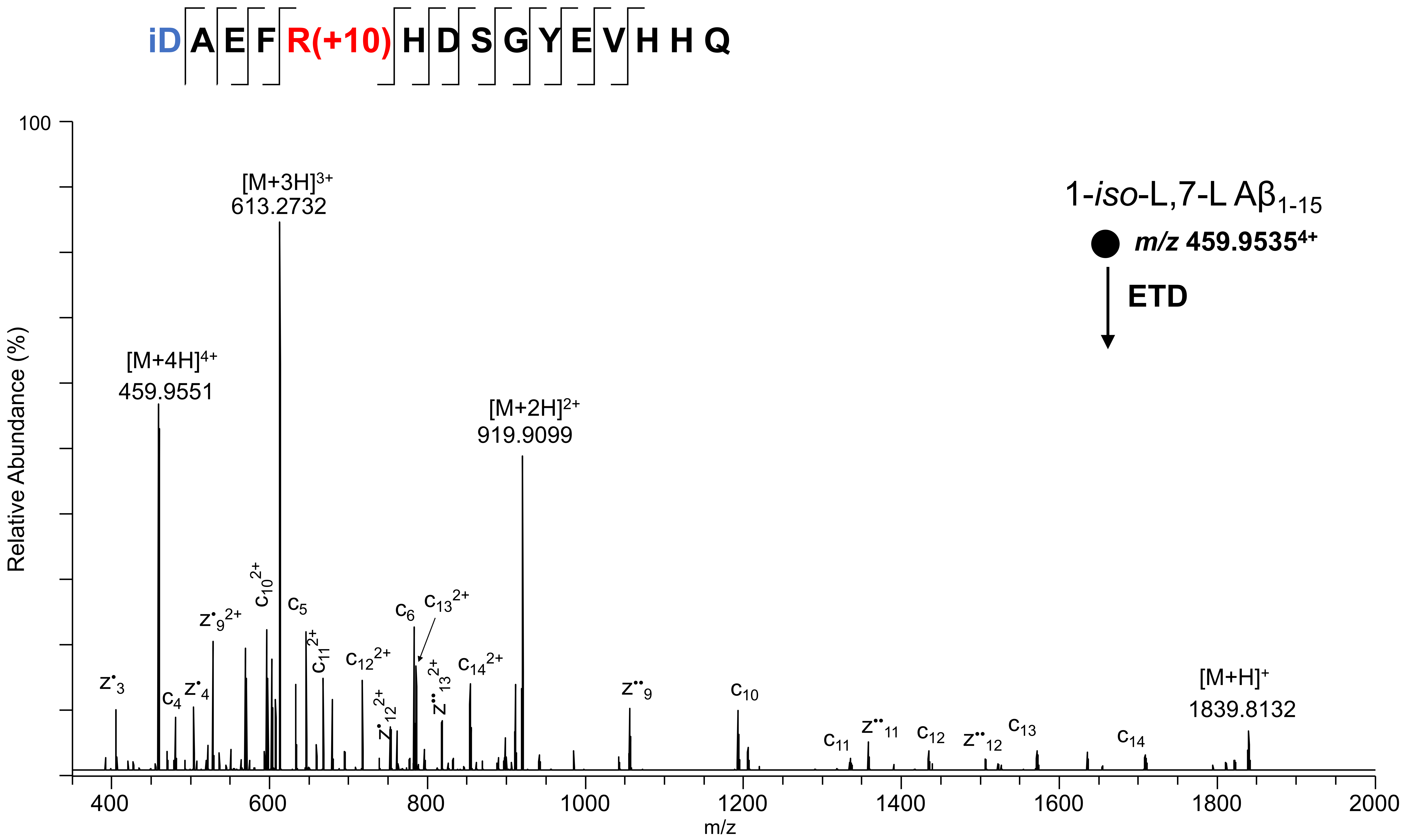

iD A E F R(+10) H D S G Y E V H H Q
1-iso-L,7-L Aβ1-15
m/z 459.9535⁴⁺
ETD
[M+4H]4+
459.9551
[M+3H]3+
613.2732
[M+2H]2+
919.9099
[M+H]+
1839.8132
z•3
c4
z•4
z•9 2+
c10 2+
c5
c11 2+
c12 2+
z•12 2+
c6
c13 2+
z••13 2+
c14 2+
z••9
c10
c11
z••11
c12
z••12
c13
c14
Relative Abundance (%)
100
m/z
400
600
800
1000
1200
1400
1600
1800
2000

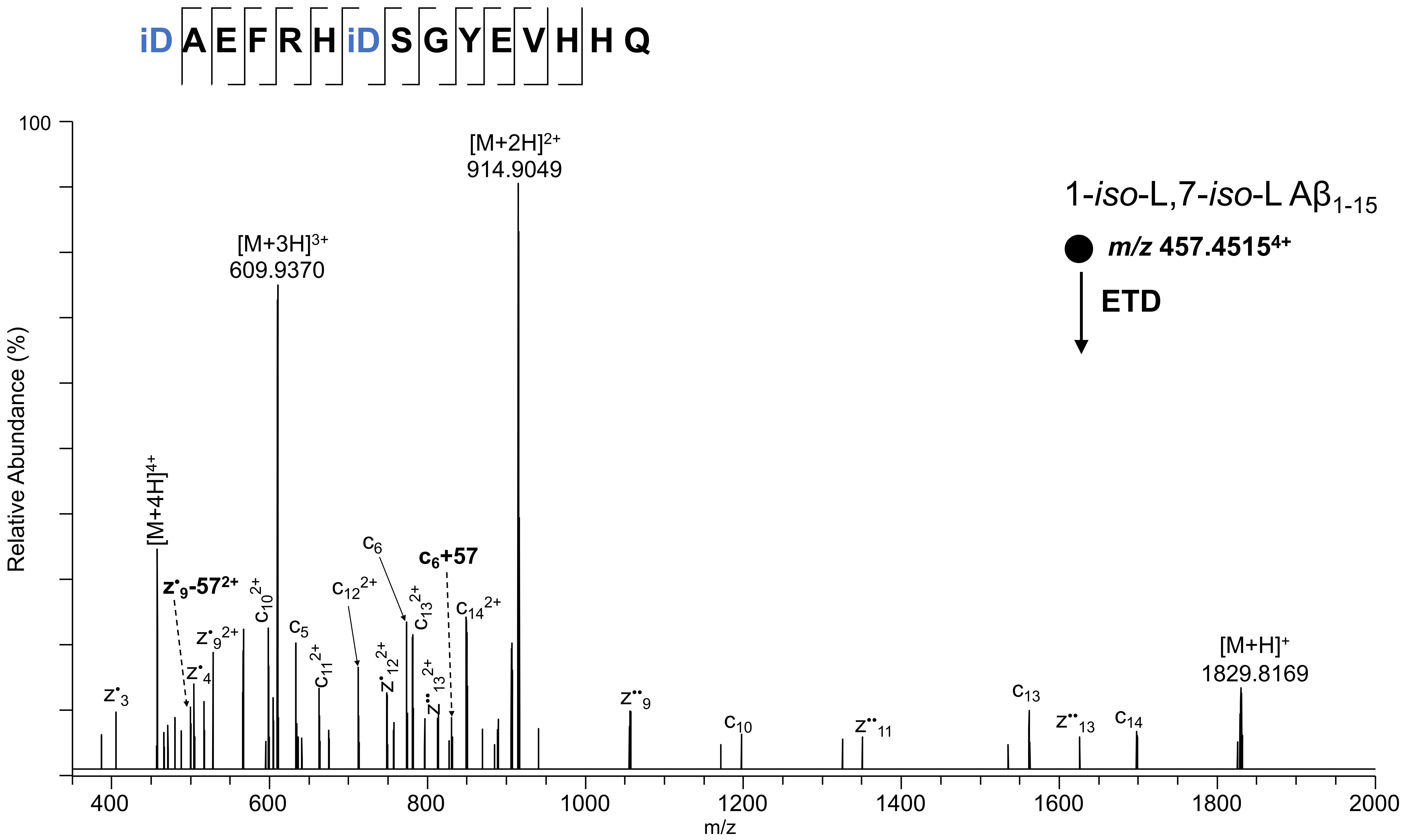

iD A E F R H iD S G Y E V H H Q
1-iso-L,7-iso-L Aβ1-15
m/z 457.4515 4+
ETD
[M+2H]2+
914.9049
[M+3H]3+
609.9370
[M+4H]4+
[M+H]+
1829.8169
z•9-57 2+
c6+57
Relative Abundance (%)
m/z

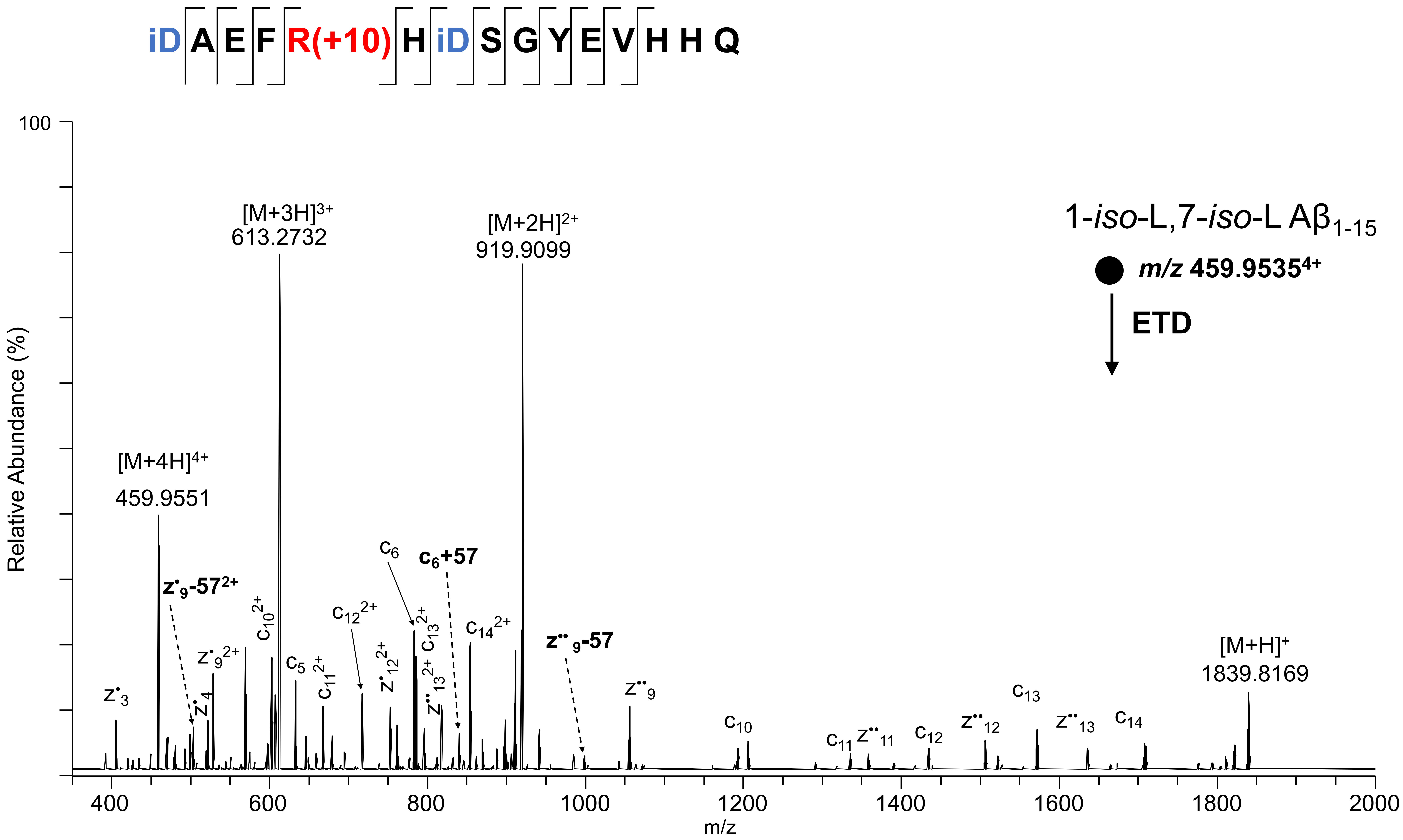
iD A E F R(+10) H iD S G Y E V H H Q
1-iso-L,7-iso-L Aβ1-15
m/z 459.9535 4+
ETD
[M+3H]3+
613.2732
[M+2H]2+
919.9099
[M+4H]4+
459.9551
[M+H]+
1839.8169
Relative Abundance (%)
m/z
100
400
600
800
1000
1200
1400
1600
1800
2000
z•3
z•9-57 2+
z•4
z•9 2+
c10 2+
c5
c11 2+
c12 2+
c6
c6+57
z•12 2+
c13 2+
z••13 2+
c14 2+
z••9-57
z••9
c10
c11
z••11
c12
z••12
c13
z••13
c14

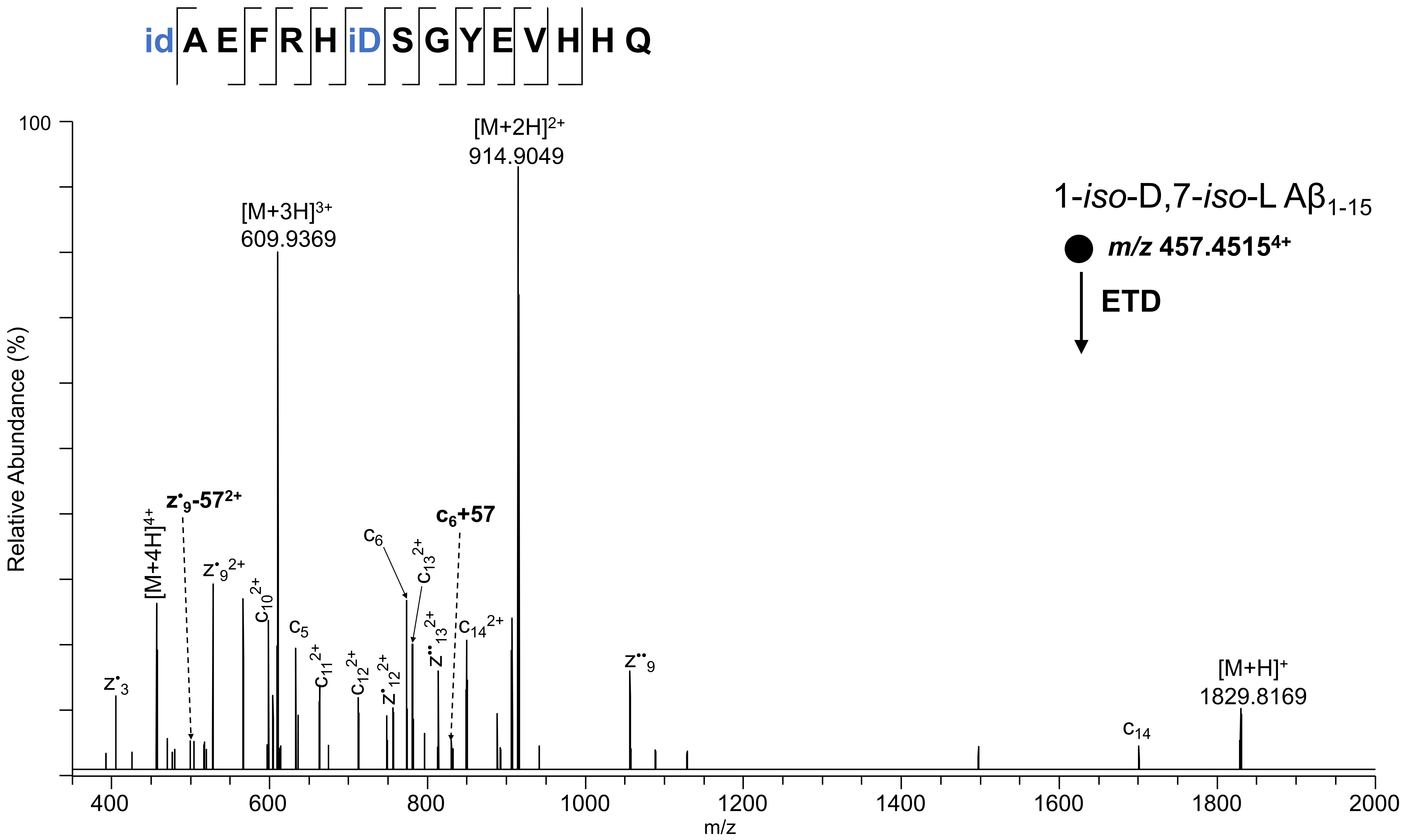

id A E F R H iD S G Y E V H H Q
1-iso-D,7-iso-L Aβ1-15
m/z 457.4515 4+
ETD
[M+2H]2+
914.9049
[M+3H]3+
609.9369
[M+H]+
1829.8169
[M+4H]4+
Relative Abundance (%)
m/z
100
400
600
800
1000
1200
1400
1600
1800
2000

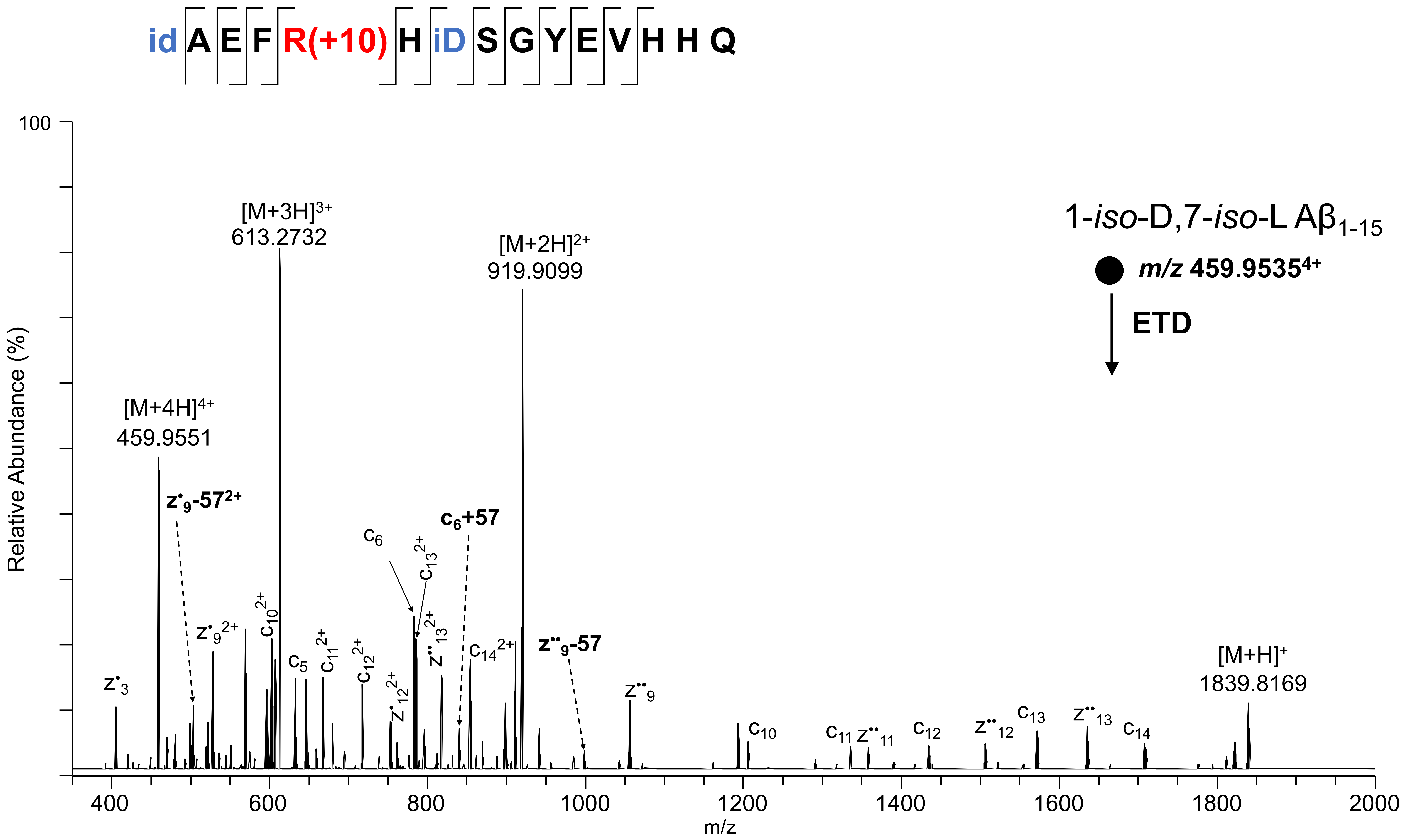

id A E F R(+10) H iD S G Y E V H H Q
1-iso-D,7-iso-L Aβ1-15
m/z 459.9535 4+
ETD
Relative Abundance (%)
m/z
[M+3H]3+ 613.2732
[M+2H]2+ 919.9099
[M+4H]4+ 459.9551
[M+H]+ 1839.8169
z•9-57 2+
c6+57
z••9-57

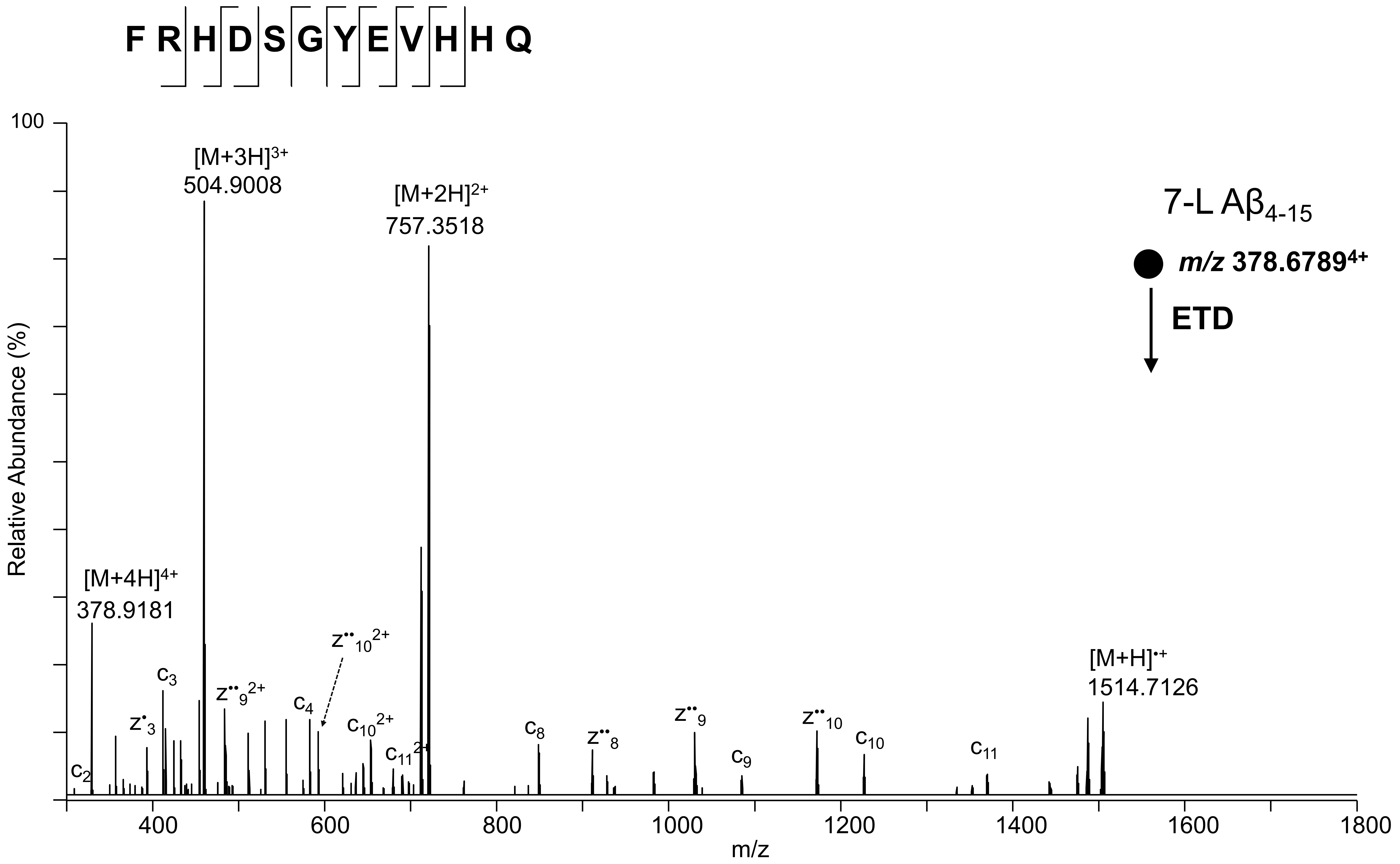

F R H D S G Y E V H H Q
7-L Aβ4-15
m/z 378.6789 4+
ETD
Relative Abundance (%)
m/z
100
[M+4H]4+
378.9181
[M+3H]3+
504.9008
[M+2H]2+
757.3518
[M+H]•+
1514.7126
c2
z•3
c3
z••9 2+
c4
z••10 2+
c10 2+
c11 2+
c8
z••8
z••9
c9
z••10
c10
c11
400
600
800
1000
1200
1400
1600
1800

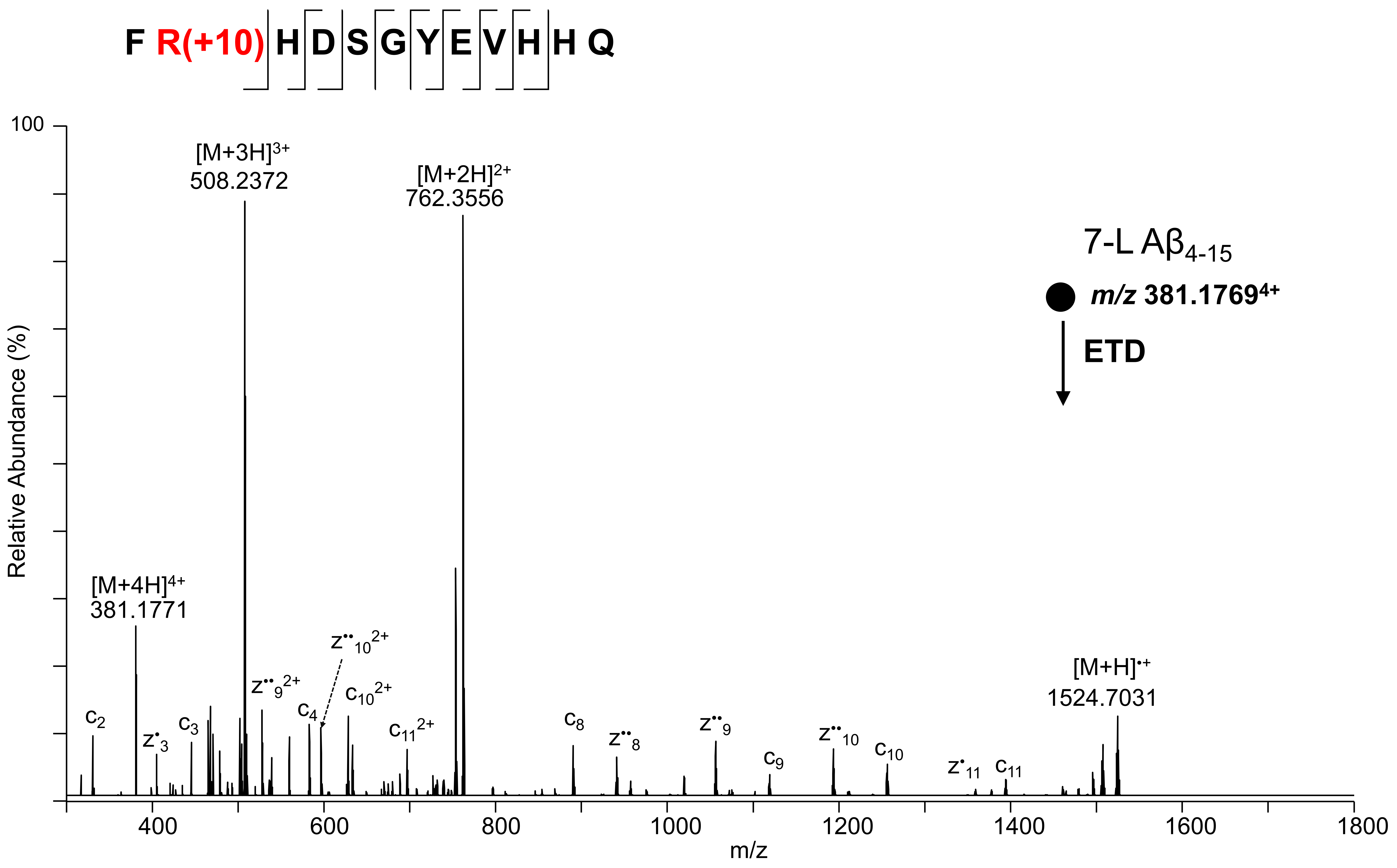

F R(+10) H D S G Y E V H H Q
7-L Aβ4-15
m/z 381.1769^4+
ETD
[M+4H]4+ 381.1771
[M+3H]3+ 508.2372
[M+2H]2+ 762.3556
[M+H]•+ 1524.7031
c2
z•3
c3
z••9 2+
c4
z••10 2+
c10 2+
c11 2+
c8
z••8
z••9
c9
z••10
c10
z•11
c11
Relative Abundance (%)
100
m/z
400
600
800
1000
1200
1400
1600
1800

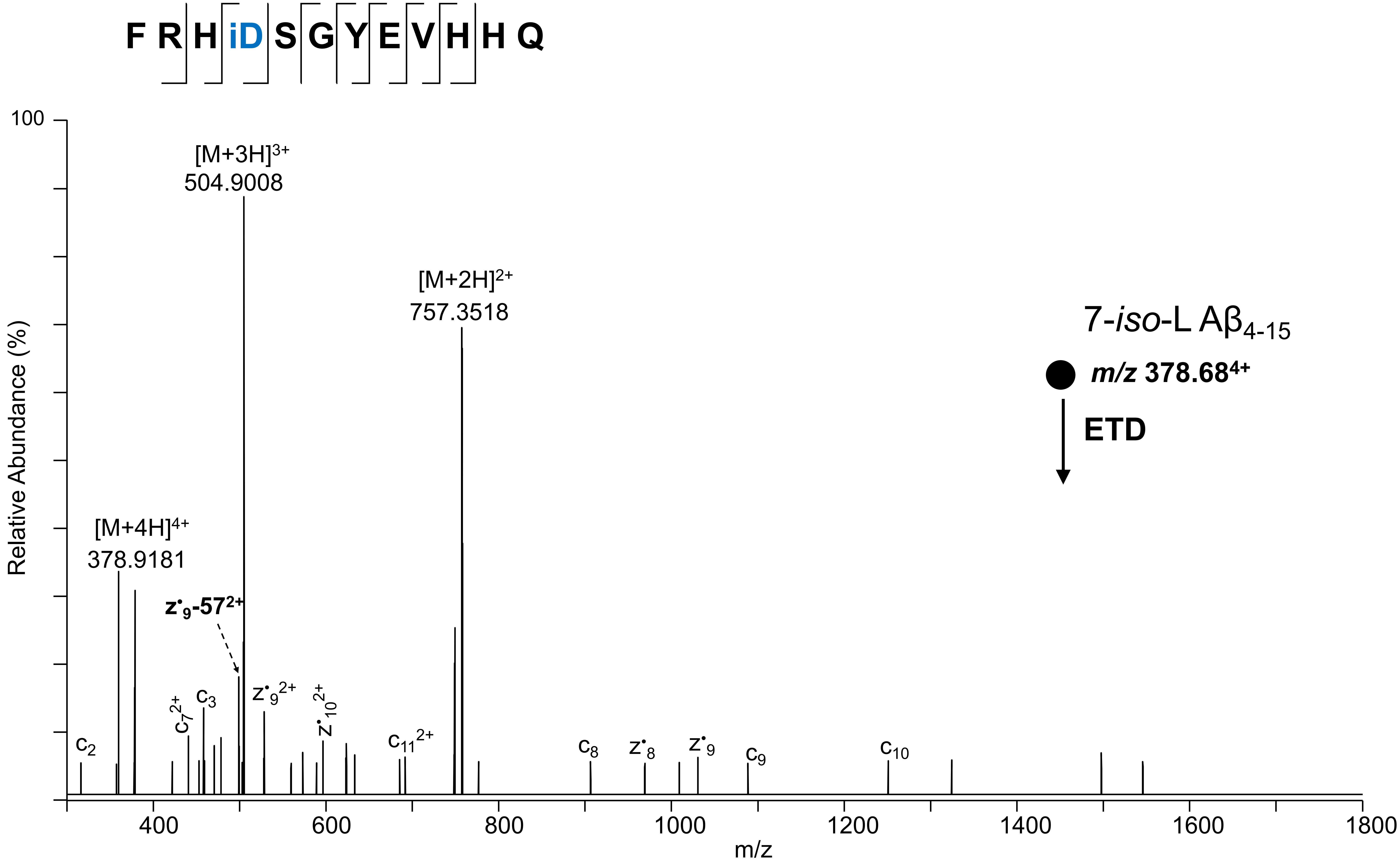

F R H iD S G Y E V H H Q
100
Relative Abundance (%)
[M+3H]3+
504.9008
[M+2H]2+
757.3518
[M+4H]4+
378.9181
z•9-57 2+
c2
c7 2+
c3
z•9 2+
z•10 2+
c11 2+
c8
z•8
z•9
c9
c10
7-iso-L Aβ4-15
m/z 378.68 4+
ETD
400
600
800
1000
1200
1400
1600
1800
m/z

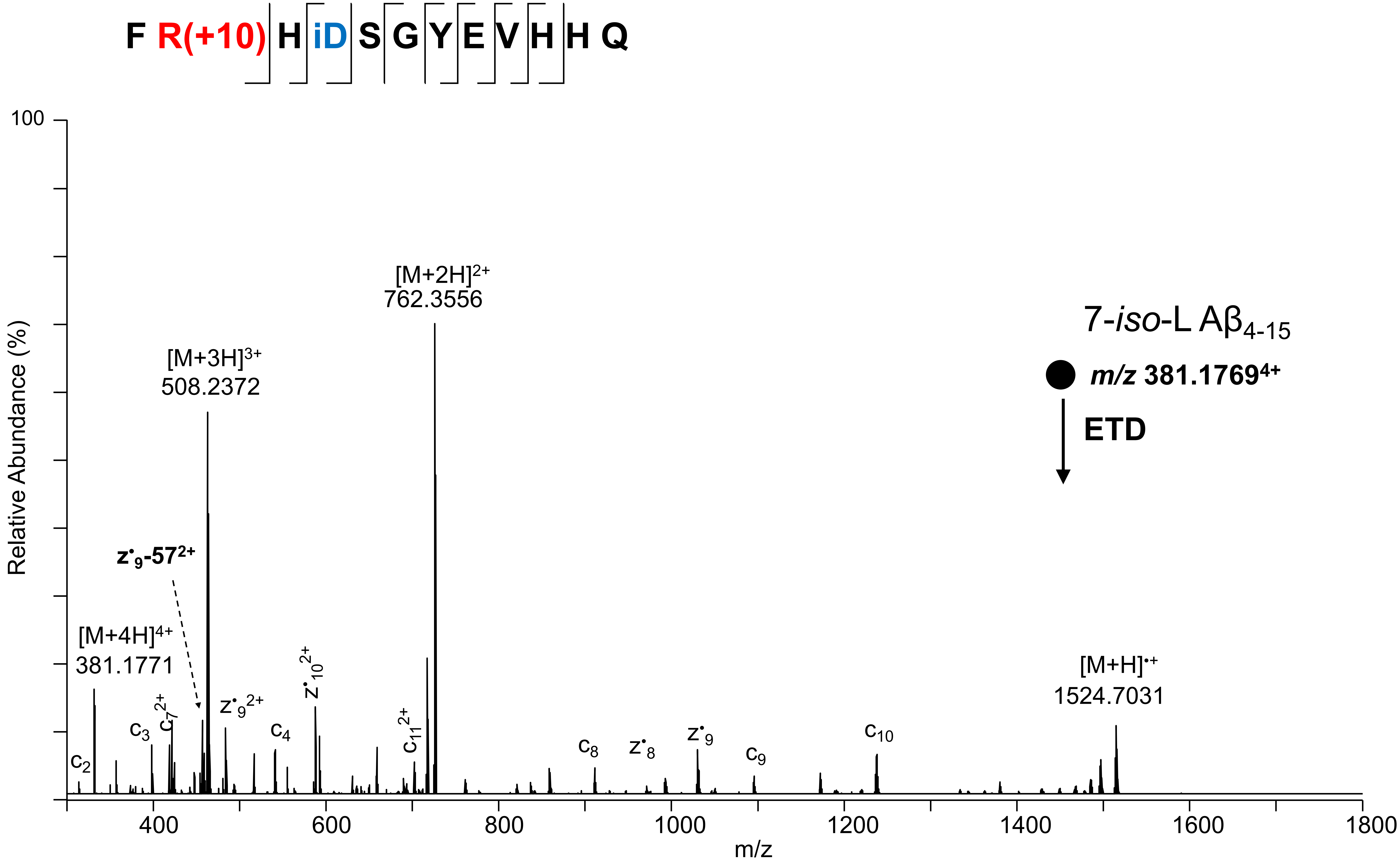

F R(+10) H iD S G Y E V H H Q
7-iso-L Aβ4-15
m/z 381.1769 4+
ETD
[M+2H]2+
762.3556
[M+3H]3+
508.2372
[M+4H]4+
381.1771
z•9-57 2+
z•9 2+
z•10 2+
c11 2+
c7 2+
c2
c3
c4
c8
z•8
z•9
c9
c10
[M+H]•+
1524.7031
Relative Abundance (%)
m/z
100
400
600
800
1000
1200
1400
1600
1800
