# Supplementary material for: Quantification of N-terminal amyloid-β isoforms reveals isomers are the most abundant form of the amyloid-β peptide in sporadic Alzheimer’s disease
Source: Brain Commun. 2021 Mar 9;3(2):fcab028. doi: 10.1093/braincomms/fcab028 (PMC8062259; doi:10.1093/braincomms/fcab028)
Supplement: fcab028_Supplementary_Data [file fcab028_supplementary_data.zip › Supplementary_material.docx]

Supporting Information

**Quantification of N-terminal Amyloid-β isoforms reveal isomers are the most abundant form of the Amyloid-β peptide** **in sporadic Alzheimer’s Disease**

Soumya Mukherjee,1 Keyla A. Perez,1 Larissa C. Lago,1 Stephan Klatt,1 Catriona A. McLean1,2, Ian E. Birchall,1 Kevin J. Barnham,1 Colin L. Masters1 and Blaine R. Roberts1,3,4*

1Florey Institute of Neuroscience and Mental Health, University of Melbourne, Melbourne, Victoria 3010, Australia

2Department of Anatomical Pathology, Alfred Hospital, Prahran, Victoria 3004, Australia

3Department of Biochemistry, Emory University School of Medicine, Atlanta, GA 30322 USA

4Department of Neurology, Emory University School of Medicine, Atlanta, GA 30322 USA

Corresponding Authors: [blaine.roberts@emory.edu](mailto:blaine.roberts@emory.edu)

Current Address:

Department of Biochemistry,

Department of Neurology,

Emory University School of Medicine,

Atlanta, GA, 30322, USA

**Nano-LC-ESI-ETD-PRM**

Electron transfer dissociation-parallel reaction monitoring (ETD-PRM) of peptides from brain digests were performed on an Orbitrap Fusion™ Lumos Tribrid™ mass spectrometer (Thermo Scientific™). 1 μg of total digested peptides or synthetic peptides in peptide loading buffer (2 % ACN, 0.05 % FA) were loaded on a trap column (5 cm Χ 75 μm internal diameter C18 column, Thermo) and separated on Acclaim PepMap RSLC column (25 cm Χ 75 μm internal diameter) by Dionex UltiMate 3000 RSLCnano liquid chromatography system (Thermo Scientific) using two mobile phases; Buffer A (0.1 % FA in water) and Buffer B (0.1 % FA in 100 % ACN). The peptides were separated using the following gradient, 0 min, 0 % Buffer B; 6 min, 3 % Buffer B; 95 min, 23 % Buffer B; 105 min, 40 % Buffer B; 110 min, 80 % Buffer B; 115 min, 80 % Buffer B; 125 min, 3 % Buffer B. Orbitrap MS1 resolution, 120,000; ion injection time (ms), 27.5; AGC, on; ETD was performed on the ions of interest in a PRM mode.

**Comparison of Neat formic acid and SISCAPA Aβ isomers enrichment**

Equal amount to total protein in lyophilized FA fractions and Na2CO3/TBS fractions from one AD patient were reduced with dithiothreitol (DTT 20 mM) at 37 °C for 30 minutes, followed by alkylation using 25 mM iodoacetamide (IAA) in the dark for another 30 minutes. The samples were then diluted to 200 μL with 100 mM TEAB buffer, pH 8.5 and digested overnight by incubation at 37 °C after adding LysN metalloprotease at enzyme:protein ratio of 1:100. The digestion reaction was quenched by adding 10 % FA to a final concentration of 0.1 %. The FA as well as the Na2CO3/TBS fractions were then spiked with only 10 μL of SIS Aβ peptides mixture (200 fmol/μL of Aβ NEP peptides) to keep the SIS peptides constant. The acidified samples were finally loaded onto an Oasis HLB μElution 96 well-plate (Waters) and purified according to manufacturer’s protocol. The desalted peptides were lyophilized before MS analysis.

The SISCAPA enrichment experiments for Na2CO3/TBS fractions were performed in a round-bottom 96-well polypropylene plates using the magnetic bead protocol as described in the method section using 1 μg of each polyclonal SISCAPA antibodies. The captured peptides were thoroughly washed followed by elution with 25 μL of 5 % acetic acid, 15 % ACN with shaking at 600 rpm and 2 min incubation.

Finally, either the neat FA digested peptides (resuspended in 25 μL of 0.05 % FA, 2.5 % ACN) or SISCAPA enriched peptides were loaded on to the LC-ESI-IMS-MS

**Supplementary Table 1.** Demographics of *post-mortem* temporal cortex with immunohistopathology of amyloid staining. The degree of pathology is assessed semi-quantitatively and indicated by: −, absent or not discernible, +, slight; ++, moderate; +++, severe.

| **ID** | **Genotype** | **Diagnosis** | **Age** | **PMI (hrs)** | **Sex** | **Sample ID** | **Plaque Aβ** | **Vessel Aβ** |
| --- | --- | --- | --- | --- | --- | --- | --- | --- |
| 03/213 | E3/E3 | AD | 80.6 | 24 | M | ***1*** | +++ | +/- |
| 03/480 | E3/E3 | AD | 70.3 | 13.5 | M | ***2*** | +++ | + |
| 04/157 | E3/E4 | HC | 68.3 | 71.5 | F | ***3*** | + | - |
| 04/041 | E3/E3 | HC | 82.5 | 22 | M | ***4*** | + | - |
| 04/107 | E3/E3 | HC | 82.5 | 57.5 | F | ***5*** | - | - |
| 04/341 | E3/E4 | HC | 60.4 | 48.5 | F | ***6*** | - | - |
| 04/686 | E2/E3 | AD | 80.6 | 67 | M | ***7*** | ++ | + |
| 05/425 | E3/E4 | AD | 94.5 | 23 | F | ***8*** | ++ | + |
| 05/321 | E3/E4 | HC | 66.9 | 10 | M | ***9*** | + | - |
| 05/684 | E3/E4 | AD | 88.6 | 58 | F | ***10*** | ++ | - |
| 06/287 | E3/E4 | AD | 99.8 | 44 | F | ***11*** | ++ | ++ |
| 06/906 | E3/E4 | AD | 87.4 | 39 | F | ***12*** | ++ | +++ |
| 06/361 | E4/E4 | AD | 92.7 | 14 | F | ***13*** | ++ | + |
| 06/849 | E2/E3 | HC | 82.7 | 27 | M | ***14*** | - | - |
| 07/104 | E3/E3 | HC | 52.3 | 52 | M | ***15*** | - | - |
| 07/674 | E3/E4 | AD | 88.4 | 11 | F | ***16*** | +++ | ++ |
| 07/678 | E3/E4 | HC | 69.6 | 71 | M | ***17*** | + | - |
| 07/743 | E3/E3 | HC | 63.9 | 68 | M | ***18*** | + | - |
| 10/044 | E4/E4 | AD | 83.5 | 27 | F | ***19*** | +++ | ++ |
| 10/305 | E3/E3 | AD | 59.7 | 4 | F | ***20*** | +++ | + |

**Supplementary Table 2.** Multiple reaction monitoring (MRM) transitionsused for Aβ SISCAPA on 6495 QQQ LC/MS (Agilent Technologies).

| **Peptide Sequence** | **Precursor Ion** | **Product Ion** | **CE (V)** | **Ret Time (min)** | **Ret Window** | **Polarity** |
| --- | --- | --- | --- | --- | --- | --- |
| DAEFRHDSGYEVHHQ.heavy | 459.953549 | 869.390041 | 17.6 | 17 | 8 | Positive |
| DAEFRHDSGYEVHHQ.heavy | 459.953549 | 761.346497 | 14.6 | 17 | 8 | Positive |
| DAEFRHDSGYEVHHQ.heavy | 459.953549 | 520.262656 | 17.6 | 17 | 8 | Positive |
| DAEFRHDSGYEVHHQ.heavy | 459.953549 | 421.194242 | 17.6 | 17 | 8 | Positive |
| DAEFRHDSGYEVHHQ.light | 457.451482 | 956.42207 | 14.6 | 17 | 8 | Positive |
| DAEFRHDSGYEVHHQ.light | 457.451482 | 869.390041 | 17.6 | 17 | 8 | Positive |
| DAEFRHDSGYEVHHQ.light | 457.451482 | 756.342363 | 14.6 | 17 | 8 | Positive |
| DAEFRHDSGYEVHHQ.light | 457.451482 | 520.262656 | 17.6 | 17 | 8 | Positive |
| DAEFRHDSGYEVHHQ.light | 457.451482 | 421.194242 | 17.6 | 17 | 8 | Positive |
| FRHDSGYEVHHQ.heavy | 381.176887 | 649.305249 | 8.7 | 10.6 | 6 | Positive |
| FRHDSGYEVHHQ.heavy | 381.176887 | 536.228144 | 5.7 | 10.6 | 6 | Positive |
| FRHDSGYEVHHQ.heavy | 381.176887 | 520.262656 | 8.7 | 10.6 | 6 | Positive |
| FRHDSGYEVHHQ.heavy | 381.176887 | 421.194242 | 8.7 | 10.6 | 6 | Positive |
| FRHDSGYEVHHQ.light | 378.674819 | 649.305249 | 8.7 | 10.6 | 6 | Positive |
| FRHDSGYEVHHQ.light | 378.674819 | 536.228144 | 5.7 | 10.6 | 6 | Positive |
| FRHDSGYEVHHQ.light | 378.674819 | 520.262656 | 8.7 | 10.6 | 6 | Positive |
| FRHDSGYEVHHQ.light | 378.674819 | 421.194242 | 8.7 | 10.6 | 6 | Positive |
| KGAIIGLMVGGVV.heavy | 611.377583 | 1104.668911 | 26.6 | 32.6 | 5 | Positive |
| KGAIIGLMVGGVV.heavy | 611.377583 | 1005.600497 | 26.6 | 32.6 | 5 | Positive |
| KGAIIGLMVGGVV.heavy | 611.377583 | 948.579034 | 19.8 | 32.6 | 5 | Positive |
| KGAIIGLMVGGVV.heavy | 611.377583 | 891.55757 | 23.6 | 32.6 | 5 | Positive |
| KGAIIGLMVGGVV.heavy | 611.377583 | 792.489156 | 22.8 | 32.6 | 5 | Positive |
| KGAIIGLMVGGVV.light | 607.370484 | 1096.654712 | 26.6 | 32.6 | 5 | Positive |
| KGAIIGLMVGGVV.light | 607.370484 | 997.586298 | 26.6 | 32.6 | 5 | Positive |
| KGAIIGLMVGGVV.light | 607.370484 | 940.564835 | 19.8 | 32.6 | 5 | Positive |
| KGAIIGLMVGGVV.light | 607.370484 | 883.543371 | 23.6 | 32.6 | 5 | Positive |
| KGAIIGLMVGGVV.light | 607.370484 | 784.474957 | 22.8 | 32.6 | 5 | Positive |
| KGAIIGLMVGGVVIA.heavy | 703.438172 | 1203.737325 | 23.2 | 35.8 | 5 | Positive |
| KGAIIGLMVGGVVIA.heavy | 703.438172 | 1104.668911 | 26.2 | 35.8 | 5 | Positive |
| KGAIIGLMVGGVVIA.heavy | 703.438172 | 1005.600497 | 29.2 | 35.8 | 5 | Positive |
| KGAIIGLMVGGVVIA.heavy | 703.438172 | 948.579034 | 28.9 | 35.8 | 5 | Positive |
| KGAIIGLMVGGVVIA.heavy | 703.438172 | 891.55757 | 28.9 | 35.8 | 5 | Positive |
| KGAIIGLMVGGVVIA.light | 699.431072 | 1195.723126 | 23.2 | 35.8 | 5 | Positive |
| KGAIIGLMVGGVVIA.light | 699.431072 | 1096.654712 | 26.2 | 35.8 | 5 | Positive |
| KGAIIGLMVGGVVIA.light | 699.431072 | 997.586298 | 29.2 | 35.8 | 5 | Positive |
| KGAIIGLMVGGVVIA.light | 699.431072 | 940.564835 | 28.9 | 35.8 | 5 | Positive |
| KGAIIGLMVGGVVIA.light | 699.431072 | 883.543371 | 28.9 | 35.8 | 5 | Positive |
| KLVFFAEDVGSN.heavy | 667.347534 | 1201.6343 | 17.6 | 28.3 | 5 | Positive |
| KLVFFAEDVGSN.heavy | 667.347534 | 1057.580808 | 20.6 | 28.3 | 5 | Positive |
| KLVFFAEDVGSN.heavy | 667.347534 | 958.512394 | 23.6 | 28.3 | 5 | Positive |
| KLVFFAEDVGSN.heavy | 667.347534 | 843.485451 | 17.6 | 28.3 | 5 | Positive |
| KLVFFAEDVGSN.light | 663.340435 | 1193.620101 | 17.6 | 28.3 | 5 | Positive |
| KLVFFAEDVGSN.light | 663.340435 | 1106.588073 | 20.6 | 28.3 | 5 | Positive |
| KLVFFAEDVGSN.light | 663.340435 | 1049.566609 | 20.6 | 28.3 | 5 | Positive |
| KLVFFAEDVGSN.light | 663.340435 | 950.498195 | 23.6 | 28.3 | 5 | Positive |
| KLVFFAEDVGSN.light | 663.340435 | 835.471252 | 17.6 | 28.3 | 5 | Positive |

**Supplementary Table 3.** N-terminal heterogeneity of Aβ peptides identified using bottom-up proteomics from sporadic Alzheimer’s brain (Formic acid fraction). Our data is consistent with previous reports.1, 2 p, Pyroglutamate.

| Aβ | Peptide Sequence | *m/z* | *z* | Error (ppm) |
| --- | --- | --- | --- | --- |
| 8-15 | SGYEVHHQ | 478.7126 | 2 | -0.4 |
| 16-25 | KLVFFAEDVG | 562.8032 | 2 | 0.5 |
| 28-40 | KGAIIGLMVGGVV | 607.3704 | 2 | -0.2 |
| 16-27 | KLVFFAEDVGSN | 663.3399 | 2 | 0.17 |
| 5-15 | RHDSGYEVHHQ | 682.808 | 2 | -0.4 |
| 28-42 | KGAIIGLMVGGVVIA | 699.4313 | 2 | 0.5 |
| 4-15 | FRHDSGYEVHHQ | 378.6749 | 4 | 0.1 |
| pGlu3-15 | pEFRHDSGYEVHHQ | 406.4328 | 4 | -0.3 |
| 2-15 | AEFRHDSGYEVHHQ | 428.6948 | 4 | 0.3 |
| 1-15 | DAEFRHDSGYEVHHQ | 457.4515 | 4 | 0.3 |
| -2-15 | KMDAEFRHDSGYEVHHQ | 522.2361 | 4 | 0.29 |

**Supplementary Figure 1**. (A) Capture Efficiency of the polyclonal anti-peptide antibodies for each peptide of interest. (B) Calibration curve for multiplexed SISCAPA assay for Aβ1-15, Aβ4-15, Aβ16-27, Aβ28-40 and Aβ28-42 along with their limit of detection (LOD, black) and limit of quantitation (LOQ, blue) for quantitative estimation in Na2CO3 and TBS fractions.

**Supplementary Figure 2.** LC-MS separation of SIS Aβ1-15 [M+4H]4+ *m/z* 459.9535 diastereomers on Agilent Advanced Bio Peptide Mapping (2.1x150 mm, 2.7 μm) reversed phase column with their single-field DTCCSN2 in 6560 DTIM-QTOF (Agilent).

**Supplementary Figure 3**. 2D-LC-IMS-MS representation for possible SIS isomers combinations of Aβ1-15 spiked into digested FA fraction extracted from AD brain.(A) endogenous [M+4H]4+ *m/z* 457.4515 (top LC panel, red) that has been spiked with mixture of SIS Aβ1-15 epimers [M+4H]4+ *m/z* 459.9535 (bottom panel, blue) is composed of 1,7-L-asp (1), 1-*iso*-L,7-L-asp (4), 1-*iso*-L,7-*iso*-L-asp (10), 1-*iso*-D,7-*iso*-L-asp (11) and 1-*iso*-D,7-*iso*-D-asp (13). The alignment of both the LC as well as the drift time (DTCCSN2) of the spiked in individual synthetic peptides reveal the most common endogenous structural isomers of Aβ1-15 are Asp-1 and Asp-7 isomer with 1-*iso*-L,7-*iso*-L-Asp (10) being the most abundant isomer. (B) Endogenous Aβ1-15 [M+4H]4+ *m/z* 457.4515 (top LC panel, red), spiked with another mixture of SIS Aβ1-15 [M+4H]4+ *m/z* 459.9535, is composed of 1-D,7-L-Asp (2), 1-*iso*-L,7-D-Asp (3) (bottom panel, dark brown) and 1-*iso*-L,7-*iso*-D-Asp (12) (bottom panel, brown). Data demonstrates that 1-*iso*-D,7-*iso*-L-Asp (11) and 1-*iso*-L,7-*iso*-D-Asp (12) are present in equal proportions in AD brain. Enantiomerization at Asp-1/Asp-7 in Aβ1-15 is minor. (C) Endogenous Aβ1-15 [M+4H]4+ *m/z* 457.4515 (top LC panel, red; same AD brain), spiked with mixture of SIS Aβ1-15 [M+4H]4+ *m/z* 459.9535, is composed of 1-D,7-D-Asp (15), 1-L,7-*iso*-L-Asp (5) and 1-*iso*-L,7-L-Asp (8) (bottom panel, blue). The highlighted (red) LC-MS region depicts co-elution of 1-L,7-L-Asp (1) and 1-D,7-D-Asp (15) with minute ΔDTCCSN2 ~ 5. This difference in IMS-MS indicates that the endogenous Aβ1-15 does not have both Asp residues enantiomerized above the LOD.

**Supplementary Figure 4.** Alignment of RT of the synthetic SIS Aβ1-15 [M+4H]4+ *m/z* 459.9535 (bottom panel) isomers in comparison with the endogenous Aβ1-15 [M+4H]4+ *m/z* 457.4515 (top red panel) ion from AD FA fraction.

**Supplementary Figure 5**. (A) LC-MS of SIS Aβ4-15 [M+4H]4+ *m/z* 381.1769 isomers with their corresponding DTCCSN2 and the 2D-LC-IMS-MS representation of Aβ4-15 [M+4H]4+ *m/z* 378.6748 (top LC panel, red) in AD brain that has been spiked with mixture of SIS Aβ4-15 isomers [M+4H]4+ *m/z* 381.1769 (bottom panel, blue) is composed of 7-L-Asp (17) and 7-*iso*-L-Asp (18). (B) LC-MS of SIS Aβ2-15 [M+4H]4+ *m/z* 431.1968 isomers with their corresponding DTCCSN2 and the 2D-LC-IMS-MS representation of AD Brain Aβ2-15 [M+4H]4+ *m/z* 428.6947 (top LC panel, red) that has been spiked with mixture of SIS Aβ2-15 isomers [M+4H]4+ *m/z* 431.1968 (bottom panel, blue) is composed of 7-L-Asp (21), 7-*iso*-L-Asp (22) and 7-*iso*-D-Asp (23).(C) 2D-LC-IMS-MS representation of AD brain AβpGlu3-15 [M+4H]4+ *m/z* 406.4328 (top LC panel, red) and their corresponding DTCCSN2 based on the LC-IMS-MS pattern of Aβ2-15 and Aβ4-15 isoforms in the formic acid fraction with Asp isomerization/racemization at Asp-7, supports three isomers are 7-L-Asp (25), 7-*iso*-L-Asp (26) and 7-*iso*-D-Asp (27).

**Supplementary Figure 6.** Nano ESI-electron transfer dissociation-parallel reaction monitoring (nESI-ETD-PRM) distinguished internal Asp isomerization in the different N-terminal isoforms of endogenous Aβ from AD brain. (A) The diagnostic z••9-572+ and c6+57 ions from LC-MS/MS of Aβ1-15 [M+4H]4+ *m/z* 457.4515 indicated at least four distinguishable *iso*-Asp-7 isomers out of six LC-MS/MS patterns, although no information regarding the Asp-1 isomerization was discernable in the PRM screening. (B) Diagnostic z••9-572+ and c5+57 ions in the LC-MS/MS pattern of Aβ2-15 [M+4H]4+ *m/z* 428.6934 indicated two *iso*-Asp-7 out of three isomers. Similarly, (C) z••9-572+ and c4+57 diagnostic ions from AβpGlu3-15 [M+4H]4+ *m/z* 406.4329 distinguished the two *iso*-Asp-7 out of the three major epimers. (D) The existence of the diagnostic z••9-572+ ion in the same nLC-MS pattern of Aβ4-15 [M+4H]4+ *m/z* 378.6791 indicates co-elution of both *iso*-Asp-7 and Asp-7-L isomers.

**Supplementary Figure 7.** (A)nESI-ETD-PRM screening of the mid-domain Aβ16-27 [M+2H]2+ *m/z* 663.3404 and two most common canonical C-terminal isoforms of Aβ28-40 [M+2H]2+ *m/z* 607.3705 and Aβ28-42 [M+2H]2+ *m/z* 699.4313 from endogenous Aβ from AD brain plaques are shown. The LC-MS/MS profiles of these three peptides along with their corresponding EIC support the existence of only one specific isoform for each.(B) 2D-LC-IMS-MS representation of endogenous Aβ16-27 [M+2H]2+ *m/z* 663.3404 spiked with SIS Aβ16-27 [M+2H]2+ *m/z* 667.3404 (co-eluting with DTCCSN2 387 Å2) demonstrates the presence of a unique mid-domain in the sporadic AD; endogenous Aβ28-40 [M+2H]2+ *m/z* 607.3705 spiked with SIS Aβ28-40 [M+2H]2+ *m/z* 611.3776 and endogenous Aβ28-42 [M+2H]2+ *m/z* 699.4284 spiked with SIS Aβ28-42 [M+2H]2+ *m/z* 703.4305.

**Supplementary Figure 8**. Scatter plots for the absolute quantitation of the N-terminus of Aβ peptides and its most abundant diastereomers in soluble TBS fraction. (A) Absolute quantitation of the total Aβ1-15 and Aβ4-15 peptides from the TBS soluble fraction and (B) their respective isomers from 3 pooled controls and 3 pooled AD brains. Significant elevation of Aβ1-15 isomers was observed in AD compared to control TBS fraction.

**Supplementary Figure 9**. Scatter plots for the absolute quantitation of the most abundant isomers of the N-terminus of Aβ peptides. Aβ peptides were compared between AD (n = 11) and C (n = 9) and analyzed via mass spectrometry. Quantitation of (A) total levels of the seven most abundant diastereomers of Aβ1-15 from the FA (top left), urea/detergent (top middle) and Na2CO3 (top right) fractions. (B) Quantitation of total levels of the three most abundant isomers of Aβ4-15 from FA (bottom left), urea/detergent (bottom middle) and Na2CO3 (bottom left) fractions.

**Supplementary Figure 10**. Immunohistochemical (IHC) staining of Aβ (plaque burden) vs the total levels of N-terminus Aβpeptides determined by mass spectrometry for the control cases used in this cohort. (A) Total Aβ1-15, (B) total levels of Aβ1-15 isomers, (C) total Aβ4-15 and (D) total levels of Aβ4-15 isomers in the amyloid rich biochemical fractions in controls (n=8) compared to their respective IHC staining.

**Supplementary Figure 11**. Correlation of longevity versus the total levels of Aβ1-15 and Aβ4-15 and percentage ratio of their respective isomers in (A) FA (n=11), (B) urea/detergent (n=11) and (C) Na2CO3 (n=9) fractions in AD brains. The total levels of Aβ1-15 and Aβ4-15 positively correlated with age at death of AD patients in FA and urea/detergent fraction, although no correlation was found for the percentage of the isomers with age at death. Two-tailed Pearson’s correlation tests were used, r correlation and *P* values are indicated on the respective tables.

**Supplementary Figure 12**. Correlation of longevity versus the total levels of Aβ1-15 and Aβ4-15 and percentage ratio of their respective isomers in (A) FA (n=8), (B) urea/detergent (n=6) and (C) Na2CO3 (n=9) fractions in control brains. No correlation was found in the any biochemical fractions for Aβ1-15 and Aβ4-15. Two-tailed Pearson’s correlation tests were used, r correlation and *P* values are indicated on the respective tables.

**Supplementary Figure 13**. (B) Scatter plots for the Aβ28-42/Aβ28-40 ratio in all the biochemical fractions. Statistically significant elevation in the Aβ28-42/Aβ28-40 ratio in AD brain tissue compared to control brains was only observed in the Na2CO3 fraction.

**Supplementary Figure 14**. Immunohistochemical (IHC) staining of Aβ (plaque burden) vs the total levels of C-terminus Aβpeptides determined by mass spectrometry for the control cases used in this cohort. Total levels/Quantification of (A) Aβ28-42 and (B) Aβ28-40 from the amyloid rich biochemical fractions of controls (n=8) compared to their respective IHC burden.

**Supplementary Figure 15.** Neuronal Aβ is primarily based on A and the loss of A correlation in the insoluble fraction of Alzheimer’s disease. Strong significant correlation between the total A and the A was found in all three biochemical fractions both in (A) Alzheimer’s disease and (B) control brains. While the levels of A (C) correlated strongly in-between the fractions in control brains, (D) there was a loss in correlation between the formic acid and urea/detergent fractions, with weak correlation between the Na2CO3 and urea/detergent fractions.

**Supplementary Figure 16.** (A) Weak correlation for Aβ28-40 with total Aβ (Aβ16-27) in the three amyloid enriched biochemical fractions extracted from the frontal cortex of AD brain, (B) while no correlation was observed in the control brains. Total Aβ28-40 significantly correlated between the biochemical fractions in (C) AD and (D) control brains.


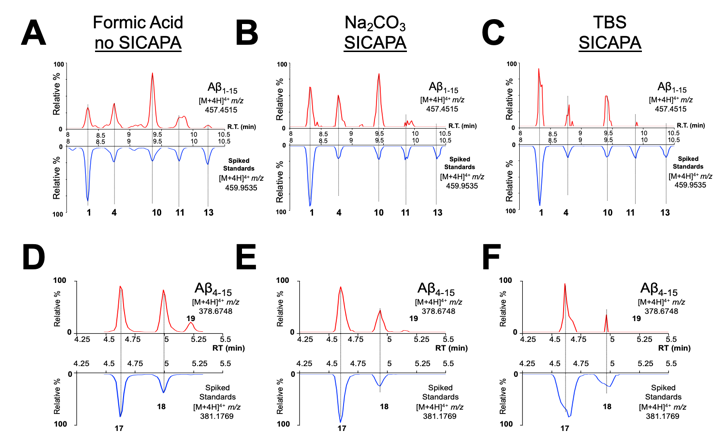


**Supplementary Figure 17**. Comparison between neat formic acid extract and SISCAPA enrichment of Aβ endogenous/synthetic isomers using polyclonal anti-Aβ antibodies from Lys-N digested formic acid fraction (A and D), Na2CO3 (B and E), and TBS fractions (C and F) for Aβ1-15 and Aβ4-15, respectively. Consistent pattern of the SIS internal standards after SISCAPA as compared to un-depleted matrix (A and D) shows equivalent affinity for the isomers by the polyclonal antibodies.

**References**

1. Portelius E*, et al.* Mass spectrometric characterization of brain amyloid beta isoform signatures in familial and sporadic Alzheimer's disease. *Acta Neuropathol* **120**, 185-193 (2010).

2. Wildburger NC*, et al.* Diversity of Amyloid-beta Proteoforms in the Alzheimer's Disease Brain. *Sci Rep* **7**, 9520 (2017).
